# miR-379 links glucocorticoid treatment with mitochondrial response in Duchenne muscular dystrophy

**DOI:** 10.1038/s41598-020-66016-7

**Published:** 2020-06-04

**Authors:** Mathilde Sanson, Ai Vu Hong, Emmanuelle Massourides, Nathalie Bourg, Laurence Suel, Fatima Amor, Guillaume Corre, Paule Bénit, Inès Barthelemy, Stephane Blot, Anne Bigot, Christian Pinset, Pierre Rustin, Laurent Servais, Thomas Voit, Isabelle Richard, David Israeli

**Affiliations:** 1 Généthon INSERM, UMR_S951, INTEGRARE research unit, Evry, 91000 France; 2 ISTEM, Inserm UMR 861, Evry, France; 3 Inserm U955-E10, IMRB, Université Paris Est, Ecole nationale vétérinaire d’Alfort, 94700 Maisons-Alfort, France; 4 Center for Research in Myology UMRS974, Sorbonne Université, INSERM, Myology Institute, Paris, France; 5 INSERM, UMR S1141, Hôpital Robert Debré, Paris, France; 6 MDUK Oxford Neuromuscular Centre, Department of Paediatrics, University of Oxford, Oxford, UK; 7 Division of Child Neurology, Centre de Références des Maladies Neuromusculaires, Department of Pediatrics, University Hospital Liège & University of Liège, Liège, Belgium; 8 NIHR Great Ormond Street Hospital Biomedical Research Centre and Great Ormond Street Institute of Child Health, University College London, London, UK

**Keywords:** Genetics, Molecular medicine, Mechanisms of disease

## Abstract

Duchenne Muscular Dystrophy (DMD) is a lethal muscle disorder, caused by mutations in the DMD gene and affects approximately 1:5000–6000 male births. In this report, we identified dysregulation of members of the Dlk1-Dio3 miRNA cluster in muscle biopsies of the GRMD dog model. Of these, we selected miR-379 for a detailed investigation because its expression is high in the muscle, and is known to be responsive to glucocorticoid, a class of anti-inflammatory drugs commonly used in DMD patients. Bioinformatics analysis predicts that miR-379 targets EIF4G2, a translational factor, which is involved in the control of mitochondrial metabolic maturation. We confirmed in myoblasts that EIF4G2 is a direct target of miR-379, and identified the DAPIT mitochondrial protein as a translational target of EIF4G2. Knocking down DAPIT in skeletal myotubes resulted in reduced ATP synthesis and myogenic differentiation. We also demonstrated that this pathway is GC-responsive since treating mice with dexamethasone resulted in reduced muscle expression of miR-379 and increased expression of EIF4G2 and DAPIT. Furthermore, miR-379 seric level, which is also elevated in the plasma of DMD patients in comparison with age-matched controls, is reduced by GC treatment. Thus, this newly identified pathway may link GC treatment to a mitochondrial response in DMD.

## Introduction

Duchenne muscular dystrophy (DMD) is a lethal muscle disorder with a prevalence of 4.78 100,000 male^1^. DMD is caused by mostly out of frame mutations in the DMD gene, located on the X chromosome, leading to a lack or drastically reduced expression of the DMD gene product, dystrophin. Deficiency of the dystrophin protein leads to the degeneration of skeletal and cardiac muscle. Whilst severely affecting motor ability, DMD also leads to premature death by respiratory or cardiac failure^2,3^. Synthetic glucocorticoids (GC) are currently the standard medication for DMD patients^4,5^. GC treatment is associated with a delayed disease progression and a reduced risk of death^6^. The therapeutic effect of GC in DMD is thought to act principally through the reduction of inflammation^7^, yet the complete mechanism of action is still unknown. However, GC drugs elicit a wide range of biological responses, including undesired side effects in DMD patients undergoing long-term treatment, such as obesity, bone fragility, pubertal delay, and short stature^4,6^. The loss of dystrophin initiates a complex pathological cascade, which includes sarcolemma destabilization, calcium influx, activation of proteases, and mitochondrial dysfunction, leading ultimately to fiber degeneration, inflammatory reaction and repeated cycles of myofiber degeneration and regeneration. The dystrophic process leads to a gradual replacement of contractile cells by fibro-adipogenic tissue and a loss of regenerative capacity. The mitochondrial defect associated with DMD pathology is characterized by reduction in expression of components in the electron transfer chain, accompanied by reduced ATP production (reviewed in^8^. The molecular mechanism(s) of mitochondrial dysfunction in DMD are, however, not entirely understood. It is thought that the calcium influx may lead to a Ca^2+^ overload within the mitochondrial matrix that may in turn result in the opening of the mitochondrial permeability transition pore (mPTP) and subsequently in mitochondrial swelling and degeneration^9,10^. However, there is an alternative hypothesis suggesting that the mitochondria dysfunction precedes the increased Ca^2+^ load within the organelle^11,12^. MicroRNAs (miRNAs) are short RNA molecules with a major influence on gene expression, due to their capacity to bind to target mRNAs. By profiling miRNA in muscle biopsies, Eisenberg and colleagues demonstrated disease-specific and distinct muscle miRNA profiles^13^. Similarly, by profiling circulating miRNA in mouse models for muscular dystrophies, we demonstrated more recently disease-specific miRNA profiles in the blood serum of these disease models^14^. Specifically for DMD, most prominent is the dysregulation of the myomiRs, which are a small number of miRNAs particularly abundant in myofibers^14–22^. However, the myomiRs are not the only dysregulated miRNA in DMD. Indeed, we recently reported such observation in a study performed in the canine GRMD (Golden retriever muscular dystrophy) model of DMD, which reproduces the human disease in many aspects^23^. The dysregulation of a large number of miRNAs in the serum of the GRMD dog model was shown, including miRNAs which are abundant in the heart (cardiomiRs) and a large number of miRNAs residing in two clusters on the genomic imprinted Dlk1-Dio3 locus^20^.

In the present study, we identified one particular member of this cluster, miR-379, as highly dysregulated in muscle biopsies of the GRMD model. We further identified a downstream GC-responding signaling pathway involving the translational factor EIF4G2 and DAPIT, a peptide associated to the mitochondrial ATP synthase. It is of note that a higher expression of DAPIT mRNA correlated with a later loss of ambulation in DMD patients^24^. More recently, DAPIT has been identified as mutated in Leigh syndrome^25^, a neurological disorder with perturbed mitochondrial functions. Thus, the signaling pathway of miR-379, EIF4G2, and DAPIT could be part of the mitochondrial dysfunction, and a target of the GC treatment effect in DMD.

## Results

### Screening for miRNA dysregulation in muscle biopsy of the GRMD dog model

**Figure 1:**
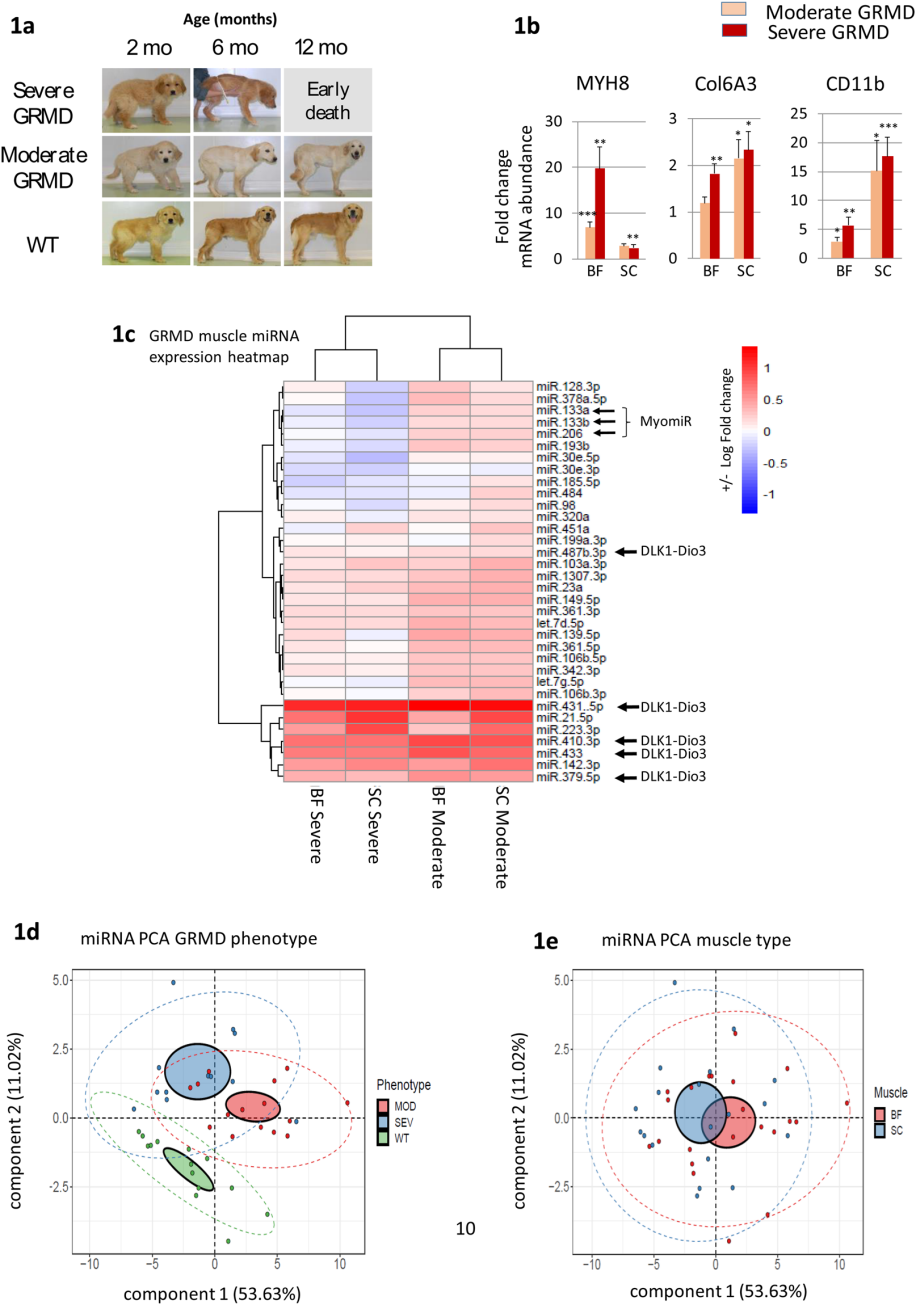
GRMD cohort and miRNA expression. 1a. Phenotype variations through ages of GRMD dogs. **1b**. Fold change of normalized mRNA abundance in muscle biopsies of 2-month old dogs (n = 9–10). CD11b: CD11 Antigen-Like Family Member, Col6a: collagen 6a3; MYH8: myosin heavy chain 8; BF: *Biceps Femoris*; SC: *Sartorius Cranialis*. **1c**. Heat map classification of miRNA dysregulation in GRMD muscle biopsies, *Biceps Femoris* (BF) and *Sartorius Cranialis* (SC) moderate and severe phenotypes. The myomiRs and miRNAs from the Dlk1-Dio3 locus are marked by black arrows. **1d**. Principal component analysis of muscle (combined SC and BF) miRNA expression in dog groups, wild type (WT), GRMD severe (SEV), and GRMD moderate (MOD). **1e**. Principal component analysis of miRNA expression in GRMD muscles (combined moderate and severe group) *Sartorius cranialis* (SC) and *biceps femoral* (BF). Circles mark the aggregation of the individuals according to their group. Small circles denoted 95% confident interval of the gravity center, while the larger discontinued-line circles surrounds 95% of the dots.

During our previous investigations on the profiling of circulating miRNAs in animal models of muscular dystrophies^14,20,26^, we identified the dysregulation of the myomiRs, the cardiomiRs and a large number of the Dlk1-Dio3 clustered miRNAs. However, whether or not these miRNAs are also dysregulated in the dystrophic skeletal muscle remains unknown. In the absence of adequate DMD muscle biopsies, we used the GRMD dog (a Golden Retriever DMD dog model), which is a highly useful animal model for DMD disease^27^. The GRMD model is characterized by a large phenotypic variability. In order to take advantage of this variability, the studied cohort was composed of phenotypically distinct 2-month old GRMD dogs: severely affected GRMD dogs (n = 10), characterized by a loss of ambulation before the age of 6 months, and moderately affected GRMD dogs (n = 9), still ambulant at 6 months of age. Healthy littermates (n = 9) were used as controls (Fig. 1a). In addition, we screened two distinct skeletal muscles of the thigh: the *Biceps femoris* (BF) and the *Sartorius cranialis* (SC). Initial molecular characterization of the samples was performed through analysis of the expression of a set of muscular dystrophy indicators: myosin heavy chain-8 (MYH8) for muscle regeneration, Col6A3 (a collagen isoform) for muscle fibrosis, and CD11b for muscle inflammation^26^ (Fig. 1b). Notably, the dysregulation of mRNA transcripts in these four sample sets correlated with the global severity of the dogs but not with the level of impairment of the two studied muscles.

We profiled in the GRMD muscles the expression of 34 miRNAs, of which three are the myomiRs. The others 31 miRNAs were previously identified as dysregulated in the serum of the mdx mouse^14^ of GRMD dogs^20^ or DMD patients (Supplemental Table 1), and were considered by us as candidates for the regulation of muscle metabolic and mitochondrial functions. Twenty-six out of the 34 miRNAs were dysregulated (FC > 1.5; p < 0.05) in at least one of the cohort groups (Table 1**)**.

**Table 1:** MiRNA expression in GRMD muscle biopsies.

miR name	Chr. Hs	Fold-change (GRMD vs WT)				p-value (GRMD vs WT)			
		Severe GRMD		moderate GRMD		Severe GRMD		moderate GRMD	
		*Biceps f*.	*Sartorius c*.	*Biceps f*.	*Sartorius c*.	*Biceps f*.	*Sartorius c*.	*Biceps f*.	*Sartorius c*.
miR-30e-3p	1	−1.46	−1.84	−1.09	−1.18		*		
miR-30e-5p	1	−1.29	−2.24	1.08	1.15				
miR-128–3p	2	1.19	−1.67	1.98	1.3			**	
miR-149-5p	2	1.37	1.39	2.45	2.61			***	***
let-7g-5p	3	−1.02	−1.25	2.01	2.02			**	**
miR-103a-3p	5	1.36	1.9	1.63	2.48		*	**	****
miR-378a-5p	5	1.03	−1.92	2.13	1.41			**	
miR-133b	6	−1.27	−1.68	1.53	1.47		*	*	
miR-206	6	−1.02	−1.42	1.58	1.55				
miR-106b-3p	7	1.07	−1.07	1.71	2.23				**
miR-106b-5p	7	1.13	1.12	1.93	1.84			**	*
miR-320a	8	1.06	−1.18	1.32	1.34				
let-7d-5p	9	1.54	1.46	2.01	2			**	**
miR-1307-3p	10	1.42	1.44	1.96	2.54			**	**
miR-139-5p	11	1.5	−1.23	3.26	3.01			***	*
miR-342-3p	14	1.3	1.14	1.93	1.89			**	*
miR-379-5p	14	2.41	2.07	3.3	2.92	**		**	***
miR-410-3p	14	5.05	5.3	8.37	7.21	**	**	**	***
miR-431-5p	14	12.41	14.89	22.09	17.61	***	**	**	***
miR-433	14	4.87	4.77	7.43	6.39	**	*	***	**
miR-487b-3p	14	1.29	1.2	1.37	1.42				
miR-193b-3p	16	−1.2	−1.61	1.9	1.72			**	
miR-484	16	−1.41	−1.3	−1.14	1.65				
miR-142-3p	17	3.1	4.15	3.09	5.12	**	*	**	**
miR-21-5p	17	4.83	10.18	2.63	8.28	**	**	**	****
miR-451a	17	−1.13	1.58	1	1.9				*
miR-133a	18	−1.28	−1.91	1.69	1.51		*	*	
miR-23a	19	1.32	1.55	2.21	2.51			**	**
miR-199a-3p	19	1.04	1.1	−1.05	1.42				
miR-185-5p	22	−1.65	−1.36	−1.18	1.3				
miR-361-3p	X	1.77	1.79	2.31	2.32		*	**	*
miR-361-5p	X	1.23	1.02	2.25	2.13			**	**
miR-98-5p	X	−1.05	−1.47	1.16	1.55				
miR-223-3p	X	3.15	8.86	1.97	6.34	*	*		**

MiRNAs are classified according chromosomal location order. Fold change GRMD/control is indicated in the middle part of the table. Respective p values are indicated *p < 0.05, **p < 0.001, ***p < 0.0005, ****p < 0.0001.

A heat map analysis of miRNA expression clustered muscle biopsies by their GRMD phenotype (severe BF with severe SC and moderate BF with moderate SC), while samples of the same muscle from the severe and moderate phenotype were separated **(**Fig. 1c**)**. Interestingly, in the cluster of the most highly dysregulated miRNAs, we found the Dlk1-Dio3 rmiRNAs, miR-379, miR-410, miR-431, miR-433 **(**Table 1 and Fig. 1c**)**, that were previously shown to be elevated in the serum of the GRMD^20^. In contrast, the myomiRs miR-133a, miR-133b and miR-206 clustered together at a reduced dysregulation level **(**Fig. 1c**)**. A PCA analysis, based on the expression data of all tested miRNAs, separated the healthy control from the GRMD dogs, and to a lesser extent the severe from the moderate GRMD phenotypes (Fig. 1d). In contrast, the Sartorius and Biceps muscle profiles remained mostly indistinguishable **(**Fig. 1e**)**. Taken together, these results demonstrated that (1) many dysregulated miRNAs in the plasma in condition of dystrophin mutations are also dysregulated in muscles; (2) globally, the miRNA upregulation is stronger in moderate versus severe GRMD muscle and (3) the Dlk1-Dio3 miRNAs (miRs −379, −410, −431, −433) are among the most highly dysregulated miRNAs in the dystrophic GRMD muscle.

### MiR-379 represses the expression of EIF4G2 and DAPIT in human myoblasts

**Figure 2:**
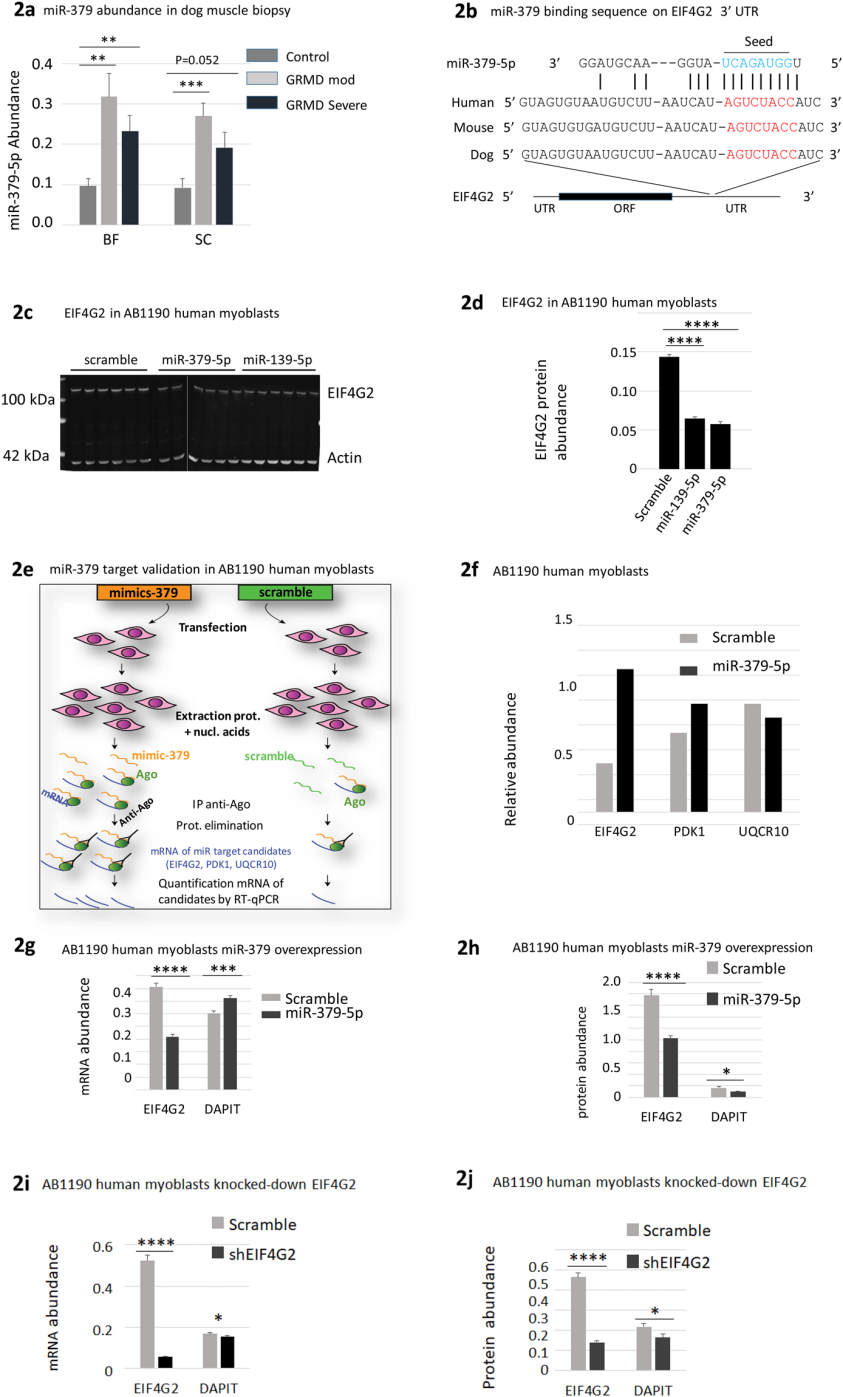
miR 379 target validation, EIF4G2 and DAPIT regulation by miR-379. 2a. miR-379 expression in GRMD muscle biopsy (a graphical presentation of miR-379 data of Table 1, (n = 9 or 10)); BF *Biceps femoris*; SC *Sartorius cranialis*. (**b**) Bioinformatics prediction of the binding site of miR-379 on EIF4G2 in human, dog and mouse. MiR-379-5p seed and complementary targets sequences are in respectively in blue and red. (**c**) EIF4G2 expression in miR-379 and miR-139 transfected AB1190 human myoblasts, shown representative results of three experiments. (**d**) Graphical presentation of (**c**). (**e)** Schematic description of the IP-Ago method for miRNA target validation. (**f**) IP-Ago enrichment for miR-379 targets sequences in AB1190 human myoblasts, shown result of one out of two independent experiments. **(g**) and (**h**) EIF4G2 and DAPIT mRNA (2 g) and protein expressions (2 h) in miR-379 overexpressing AB1190 myoblasts. **(i**,**j**) EIF4G2 and DAPIT mRNA (2i) and protein expressions (2j) in shEIF4G2 overexpressing AB1190 myoblasts.

To our knowledge, the Dlk1-Dio3 miRNAs dysregulation is yet to be investigated in the context of muscular dystrophy. We therefore sought to select one Dlk1-Dio3 miRNAs for a detailed investigation. We decided to focus on miR-379, which was reported to be GC responsive in the liver^28^. This point is of interest considering the influence of GC treatment on the DMD phenotype. In addition, miR-379 is reported to be a muscle-specific stem-cell miRNA^29^, thus potentially being involved in muscle regeneration, which is an important aspect of muscular dystrophies. A graphical representation of miR-379 expression in the GRMD BF and SC muscles is shown in Fig. 2a, demonstrating high dysregulation (except in the SC muscle, severe group, FC = 2.1, p = 0.052). In addition, the upregulation of miR-379 was found in the *tibialis cranial* muscle of 3-month old GRMD (non-significance due to the small number of biopsies), while, in cardiac left ventricles, miR-379 was not dysregulated **(**supplemental Fig. 1).

We used Diana-TarBase^30^ and TargetScanHuman^31^ for a bioinformatics prediction of miR-379 targets. Among the highest bioinformatics scores, we noticed EIF4G2, which has a binding site for miR-379-5p in its 3’ UTR (Fig. 2b**)**. EIF4G2 (also designated  DAP5 or NAT1), is a translation factor proposed to promote cap-independent IRES-driven protein translation under cellular stress conditions^32^. In addition, EIF4G2 has been shown to be required for early mouse embryo development^33^, and in a recent study, also shown to promote a mitochondrial shift of glycolytic to oxidative phosphorylation metabolism and subsequently the differentiation capacity of human ES cells^34^. Interestingly, we noted also that miR-139-5p, which is another GRMD dysregulated miRNA (Table 1) was predicted to target EIF4G2 (Supplemental Fig. 2).

To investigate whether this protein is a miR-379 target, human immortalized myoblast AB1190 cells^35^ were transfected with miR-379 and miR-139. A western blot analysis confirmed the prediction by demonstrating the downregulation of EIF4G2 in the transfected myoblasts, not only by miR-379 but also, to a very similar level, by miR-139 **(**Fig. 2c,d). We then used immunoprecipitation of the Argonaut protein (IP-Ago) method (described schematically in Fig. 2e) in order to validate the direct targeting of EIF4G2 by miR-379. After transfection of AB1190 myoblasts by miR-379, an anti-Argonaut antibody was used for the isolation of the RISC complex. The immunoprecipitated samples were analyzed by an RT-qPCR analysis using the PDK1 gene as a positive control, because it is an experimentally confirmed *bona fide* miR-379 target^36^, and the UQCR10 gene as a negative control, since it has no miR-379 recognition site. Enrichment for PDK1 and EIF4G2, but not for UQCR10, confirmed the targeting in myoblasts of EIF4G2 by miR-379-5p (Fig. 2f).

After survey of the literature, we noticed that DAPIT, a mitochondrial protein (encoded by the *USMG5* gene), was identified amongst potential translational targets of EIF4G2 in human ES cells (Supplemental material^34^). Importantly, DAPIT was shown to be expressed nearly 5 fold higher in DMD patients with late, as opposed to early loss of ambulation^24^. We hypothesized thus that miR-379, EIF4G2, and DAPIT may constitute a GC-responsive dysregulated pathway in DMD. We investigated whether the expression of DAPIT could be affected by miR-379. AB1190 human myoblasts were transfected by miR-379, and the mRNA and protein levels EIF4G2 and DAPIT quantified. Despite a slight upregulation of DAPIT mRNA expression in miR-379 transfected myoblasts (Fig. 2g, FC = 1.20), the protein level of DAPIT was significantly downregulated (Fig. 2h, FC = −1.69), supporting thus the hypothesis that within myoblasts DAPIT might be a translational target of EIF4G2. A miR-379-5p binding site could not be identify in the DAPIT UTRs and our bioinformatics analysis did not predict the possibility of a direct targeting of DAPIT transcript by miR-379. Yet, it could be hypothesized that DAPIT expression is somehow affected directly by miR-379-5p, rather than indirectly via translational control of EIF4G2. Yet, to validate the hypothesis that in myogenic cells DAPIT is a translational target of EIF4G2, rather than a direct target of miR-379, we transfected AB1190 cells by shRNA against EIF4G2. The mRNA level of DAPIT was slightly downregulated (Fig. 2i, FC = −1.22), and more clearly downregulated was its protein level (Fig. 2j, FC = −1.34).

### Myogenic expression of miR-379 EIF4G2 and DAPIT

**Figure 3:**
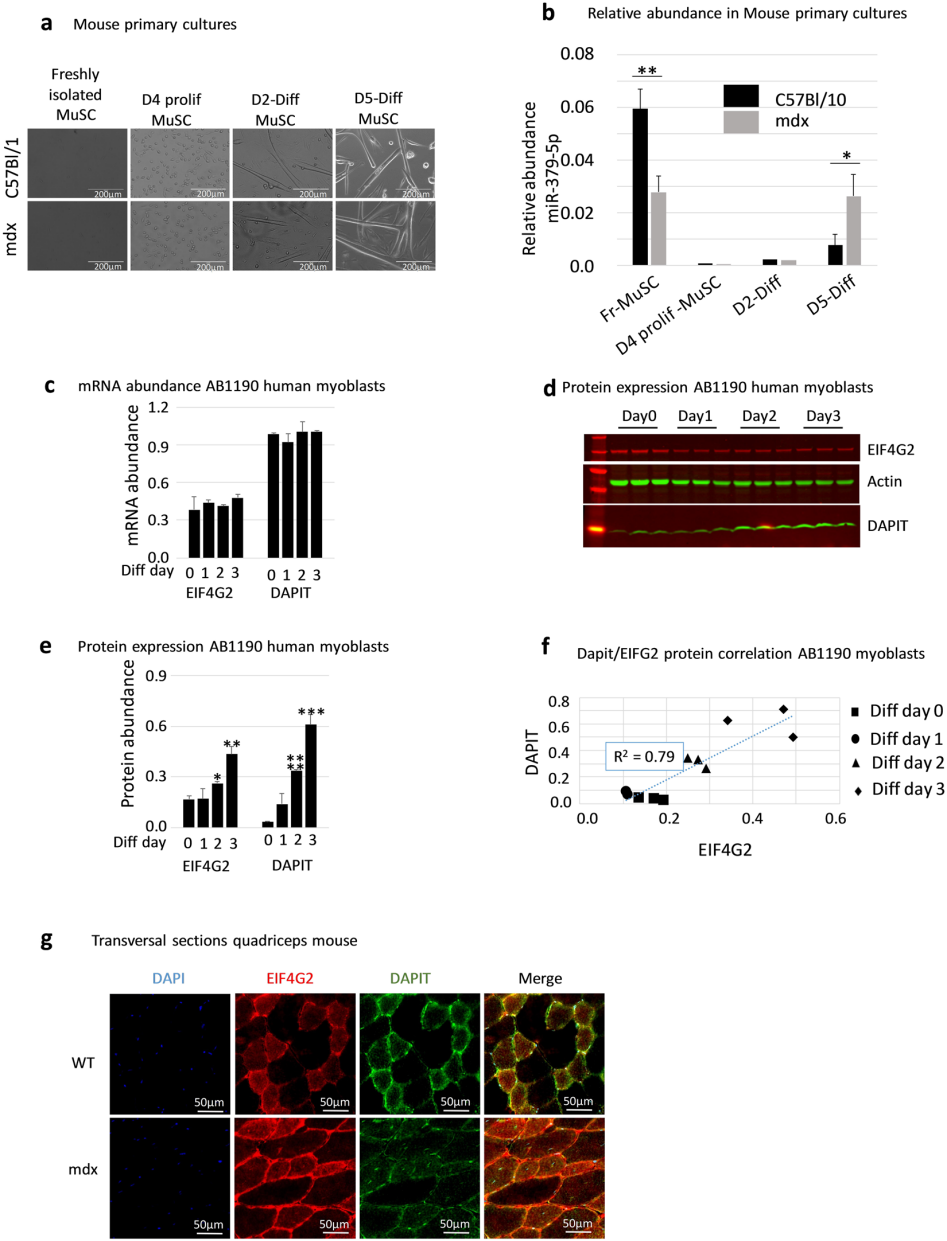
Myogenic expression of miR-379, EIF4G2 and DAPIT. **3a**. Images of freshly isolated, proliferating and differentiated muscle stem cells (MuSC). Scale bar = 200 µm. (**b**) miR-379 relative abundance in freshly isolated Fr-MuSC; Day 4 proliferation MuSC (D4 prolif MuSC), day 2 and 5 of differentiation of the same cells. (**c**) to **(e**) EIF4G2 and DAPIT mRNA **(c)** and protein **(d,e)** expressions during AB1190 human myoblasts proliferation (day 0) and differentiation (Days 1, 2 and 3). (**f**) Correlation of DAPIT to EIF4G2 protein expression in AB1190 myoblasts during proliferation (Day 0) and differentiation (Days 1, 2 and 3). Dotted line = linear regression. (**g**) immunofluorescence detection of EIF4G2 and DAPIT in transversal sections of the quadriceps muscle of adult mdx and C57Bl/10 mouse.

The skeletal muscle is a complex tissue, composed of a wide array of cell types. The origin of miRNA dysregulation in the dystrophic muscle could be of a myogenic or of a non-myogenic linage. To investigate miR-379 expression pattern in the myogenic lineage, muscle satellite cells were sorted from the hind limb muscles of the mdx and wt control mice. Satellite cells were cultured for 4 days in proliferative media (D4 proliferation) and then for additional 5 days in differentiation condition (D5 differentiation). Mir-379 expression was quantified by quantitative PCR **(**Fig. 3a,b). In agreement with a recent publication^37^, we detected a high-level of miR-379 expression in freshly isolated satellite cells from the control wt mouse, which was sharply downregulated upon myoblast proliferation **(**Fig. 3b**)**. Unexpectedly, in the freshly isolated satellite cells, miR-379 expression was lower in mdx compared to that in the wt mouse. We then hypothesized that differential expression pattern of miR-379 between mdx and control may evolve through differentiation. The overall morphology of the day 2 and 4 differentiated myotubes was similar between mdx and control mice-derived cultures (Supplemental Fig. 3**)**. However, upon *in vitro* differentiation, we detected a significantly higher level of miR-379 in mdx than in the wt control (Fig. 3a,b). Taken together, these results demonstrate that miR-379 is expressed in the myogenic lineage, at a high level in satellite cells (lower in mdx than in the control), downregulated upon satellite cells activation and proliferation, and increasingly expressed upon myogenic differentiation, to a higher level in mdx than in the control mouse. EIF4G2 was reported to promote differentiation of ES cells^34^ and during embryonic development^33^. Accordingly, we hypothesized that EIF4G2 and DAPIT may promote myogenic differentiation. To explore this aspect, we investigated the expression patterns of EIF4G2 and DAPIT in human AB1190 myoblasts during proliferation and differentiation. RNA levels of both genes were unchanged during these two stages (Fig. 3c). By contrast, the protein levels of both genes rose sharply on proliferation phase and over 3-differentiation days (Fig. 3d,e). EIF4G2 and DAPIT protein expressions were highly correlated over this period (Fig. 3f), indicating a possible translation control of DAPIT by EIF4G2. We used muscle biopsy of the adult mouse for the detection of EIF4G2 and DAPIT expression *in s*itu, and identified in transversal sections a mosaic expression pattern for both proteins. Importantly, enriched expression of the two proteins was detected in the same myofibers **(**Fig. 3g**)**, which is in agreement with translation control of DAPIT by EIF4G2.

### EIF4G2 and DAPIT are required for myogenic differentiation

**Figure 4:**
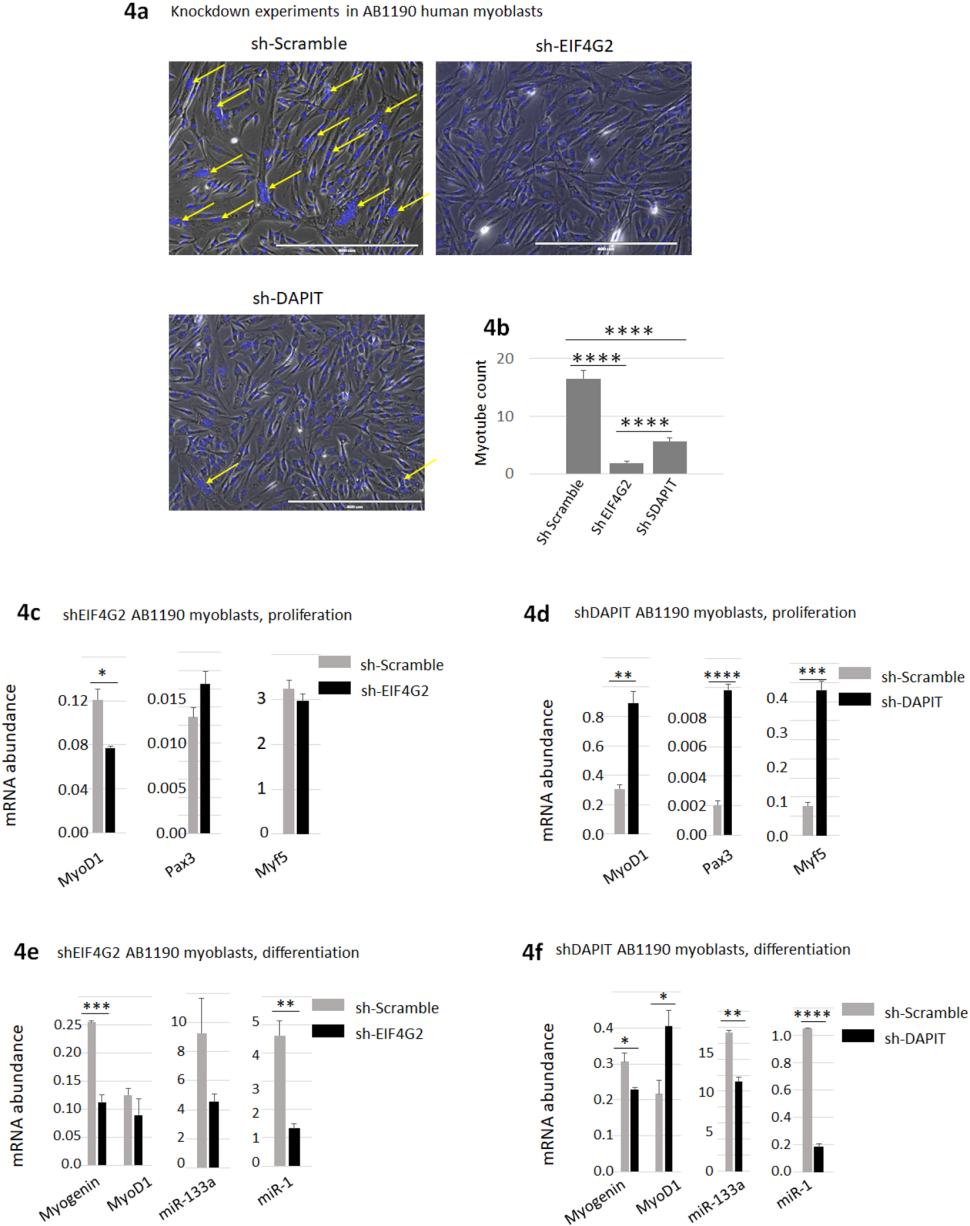
Myogenic effect of inhibition of EIF4G2 and DAPIT. (**a**,**b**) Knocking down of EIF4G2 and DAPIT expression delays differentiation of AB1190 myoblast. AB1190 myoblast were treated by shRNA (shScramble, shEIF4G2, shDAPIT). Cells were differentiated for 4 days. Myotubes containing 6 or more nuclei (yellow arrows in representative images in 3 h) were counted (presented graphically in **b**). (**c**,**d**) Muscle regulatory factors (MRF) expression in AB1190 myoblast with knocked-down expression by shRNA of EIF4G2 **(c)** and DAPIT **(d)**. (**e**,**f**) Transcriptional expression of genes associated with myogenic differentiation in shRNA knock-down EIF4G2 (**e**) and DAPIT (**f)**.

To explore further the participation of EIF4G2 and DAPIT in myogenic differentiation we investigated the effect of their inhibition. AB1190 myoblasts were transfected by shRNA to knockdown the expression of EIF4G2, or DAPIT, and then shifted to differentiation medium. It is of significance that the knockdown of either gene resulted in an inhibition of myotube formation in the cultured myoblasts (Fig. 4a,b). To understand the molecular basis of this inhibition, we quantified the expression of myogenic factors. We detected only moderately reduced MyoD expression in the proliferating AB1190 myoblasts when EIF4G2 was knocked down (Fig. 4c). In contrast, knocking down DAPIT expression resulted in a strong induction of MyoD1, Pax3 and Myf5 (Fig. 4d). A higher modification of gene expression was detected under differentiation conditions. The expression of the myogenic differentiation-associated factors myogenin, miR-1 and miR-133 was down regulated sharply upon knocking down of EIF4G2 **(**Fig. 4e**)** and DAPIT **(**Fig. 4f**)** while MyoD expression was unchanged by knocking down EIF4G2, and upregulated by knocking down DAPIT expression. This last observation would indicate a stronger requirement of EIF4G2 rather than that of DAPIT on myotube formation. Thus, knocking down the expression of either EIF4G2 or DAPIT during myoblast differentiation repressed myotube formation, as demonstrated by the phenotype and the downregulation of differentiation-associated gene expression. These results demonstrate that the expression of DAPIT is increased in correlation to the level of EIF4G2, both proteins are co-expressed in the same myofibers in the adult muscle and are required for myogenic differentiation.

### DAPIT expression and biological activity in the skeletal muscle

**Figure 5:**
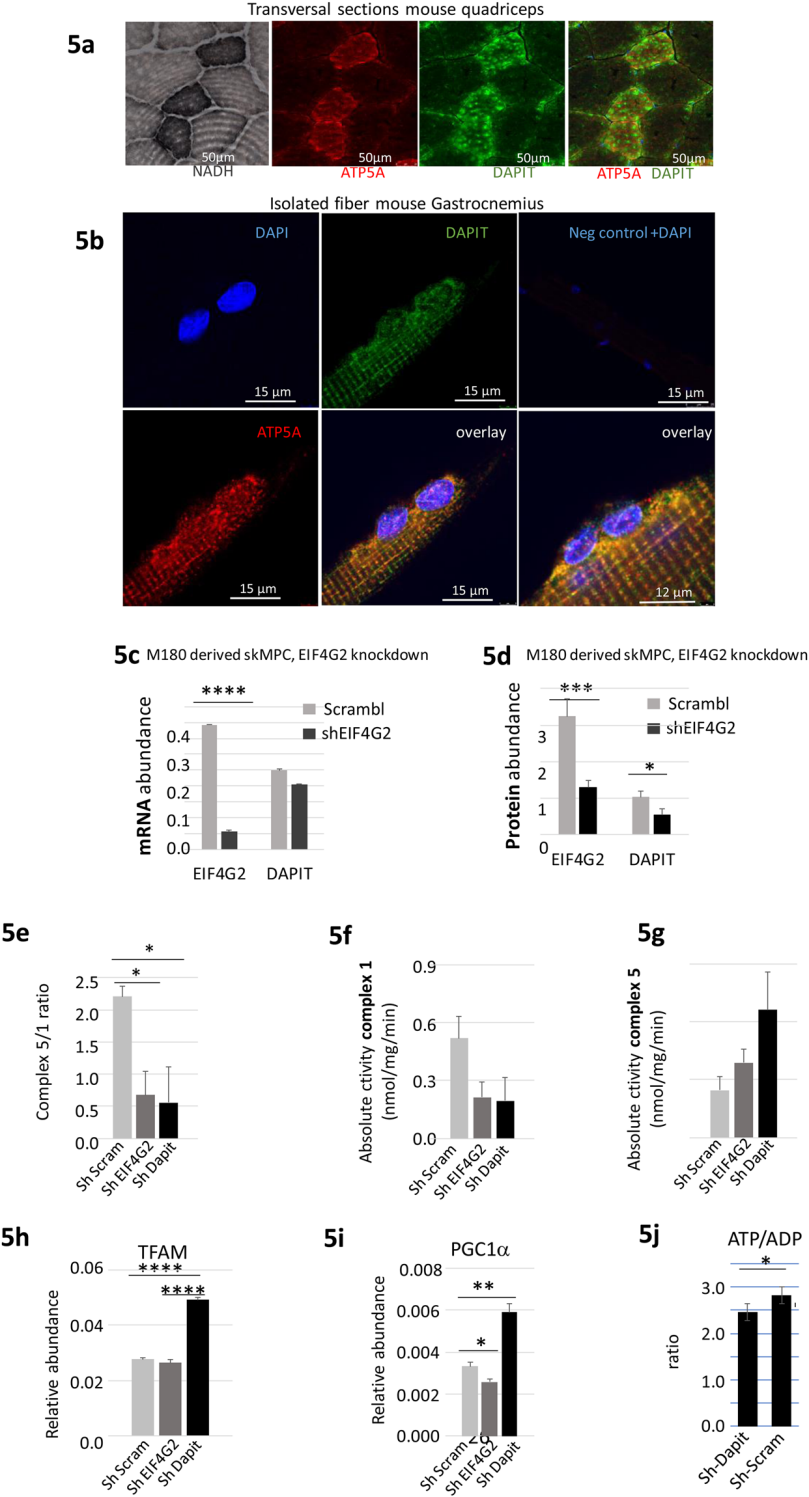
DAPIT IHC localization, mitochondrial functional studies in M180 iPS cells. (**a**) Co-localization of DAPIT with ATP5a (mitochondrial complex 5) into oxidative fibers (NADH positive) in transversal section of quadriceps muscle from adult C57Bl/10 mouse. **(b**) Co-localization DAPIT and ATP5a in single fibers isolated from the gastrocnemius of adult C57Bl/10 mouse. (**c**,**d**) EIF4G2 and DAPIT mRNA **(c)** and protein expressions (**d**) in EIF4G2 and DAPIT knocked-down M180 iPS-derived skeletal myoblasts (skMPC). (**e,f**,**g**) Mitochondrial complex 5 to 1 activity ratio **(e)** and absolute activity **(f**,**g)** in EIF4G2 and DAPIT knocked-down skMPC. **(h**,**i**) TFAM (**h**) and PGC-1α (**i**) expression in knocked-down skMPC. **(j**) ATP to ADP ration in DAPIT knockdown (sh-DAPIT) versus control (sh-Scramble) and untreated AB1190 (n = 4, in 3 independent experiments) *in vitro* differentiated myotubes.

DAPIT is reported to be a mitochondrial complex 5 ATP synthase associated peptide^38–40^. In C2C12 myoblasts, DAPIT is localized on the mitochondria, and a higher DAPIT expression can be observed in mouse oxidative myofibers, as opposed to that found in glycolytic myofibers^41^. In agreement, we confirmed a mosaic expression pattern in the mouse gastrocnemius muscle, with enriched DAPIT expression in oxidative fibers where it co-localized with the mitochondrial ATP synthase ATP5a subunit, and the oxidative enzyme Nicotinamide adenine dinucleotide (NADH) (Fig. 5a). The co-localization of DAPIT with ATP5a was confirmed in single fibers that were isolated from the gastrocnemius muscle **(**Fig. 5b).

Previous investigation demonstrated that *in vitro* knockdown of DAPIT expression in Hela cells resulted in reduced ATP production^42^. In addition, mutations in DAPIT were identified in Leigh syndrome, a severe neurological disorder characterized by a mitochondrial dysfunction^25,43^. We thus hypothesized a mitochondrial malfunction in muscle cells with reduced DAPIT expression. In transformed cell lines, mitochondrial physiology often changes into a glycolytic metabolism^44,45^. Thus, transformed cell lines might be unsuitable for mitochondria physiological investigations. In contrast, normal mitochondrial physiology is essential for the maintenance of pluripotency and the differentiation potential of pluripotent stem cells^45,46^, which makes iPS cells a better choice for mitochondrial functional analyses. We therefore selected an iPS-based experimental system for the investigation of mitochondrial functions. The iPS M180 cells were derived from human muscle biopsies and have the potential of mesodermal engagement and myogenic differentiation^47^. M180 iPS cells were transfected by shRNA against EIF4G2 (Fig. 5c,d). Following downregulation of EIF4G2, a significant downregulation of DAPIT was detected only at the protein level (DAPIT FC = −1.87, Fig. 5d) but not at the RNA level. This result confirms that, similarly to the AB1190 myoblasts, EIF4G2 controls the translation of DAPIT in M180 cells as well.

We then tested the activity of the mitochondrial complexes 1 and 5, and found that knocking-down of either EIF4G2 or DAPIT in skeletal muscle precursor cells derived from the M180 iPS cells (skMPC), resulted in a decreased ratio of complex 5 to complex 1 (C5/C1) activity **(**Fig. 5e). This decreased ratio C5/C1 was caused by conjoint reduced complex-5 and increased complex 1 activities (Fig. 5f,g respectively). Increased complex 1 activity could be explained by the increased density of mitochondria in the treated cells, possibly due to compensatory mitochondria biosynthesis. In fact, we identified the upregulation of the mitochondrial transcription regulators TFAM and PGC1α (Fig. 5h,i respectively) in the shDAPIT-treated skMPCs. Such increased of TFAM and PGC1α levels was not detected in the shEIF4G2 treated skMPCs. This divergence between the mitochondrial outcomes of knocking down EIF4G2 versus DAPIT may result from the effect of EIF4G2 on the translation of other mitochondrial Oxphos-related proteins in addition to DAPIT^35^. Taken together, it is possible that knocking down DAPIT expression in the skMPCs resulted in reduced complex 5 activity and compensatory mitochondrial biosynthesis.

Next, we investigated the effect of reduced DAPIT expression in skeletal myotubes on ATP production. DAPIT expression was knocked down in AB1190 myotubes. ATP to ADP ratio (ATP/ADP) was significantly reduced in sh-DAPIT as compared to sh-Scramble **(**p < 0.043, Fig. 5j**)**, indicating that DAPIT knockdown lowered the myotubes ATP production. This data support the hypothesis that reduced DAPIT expression in skeletal myoblasts results in reduced ATP synthesis.

### The pathway of miR-379, EIF4G2 and DAPIT is glucocorticoid responsive

**Figure 6:**
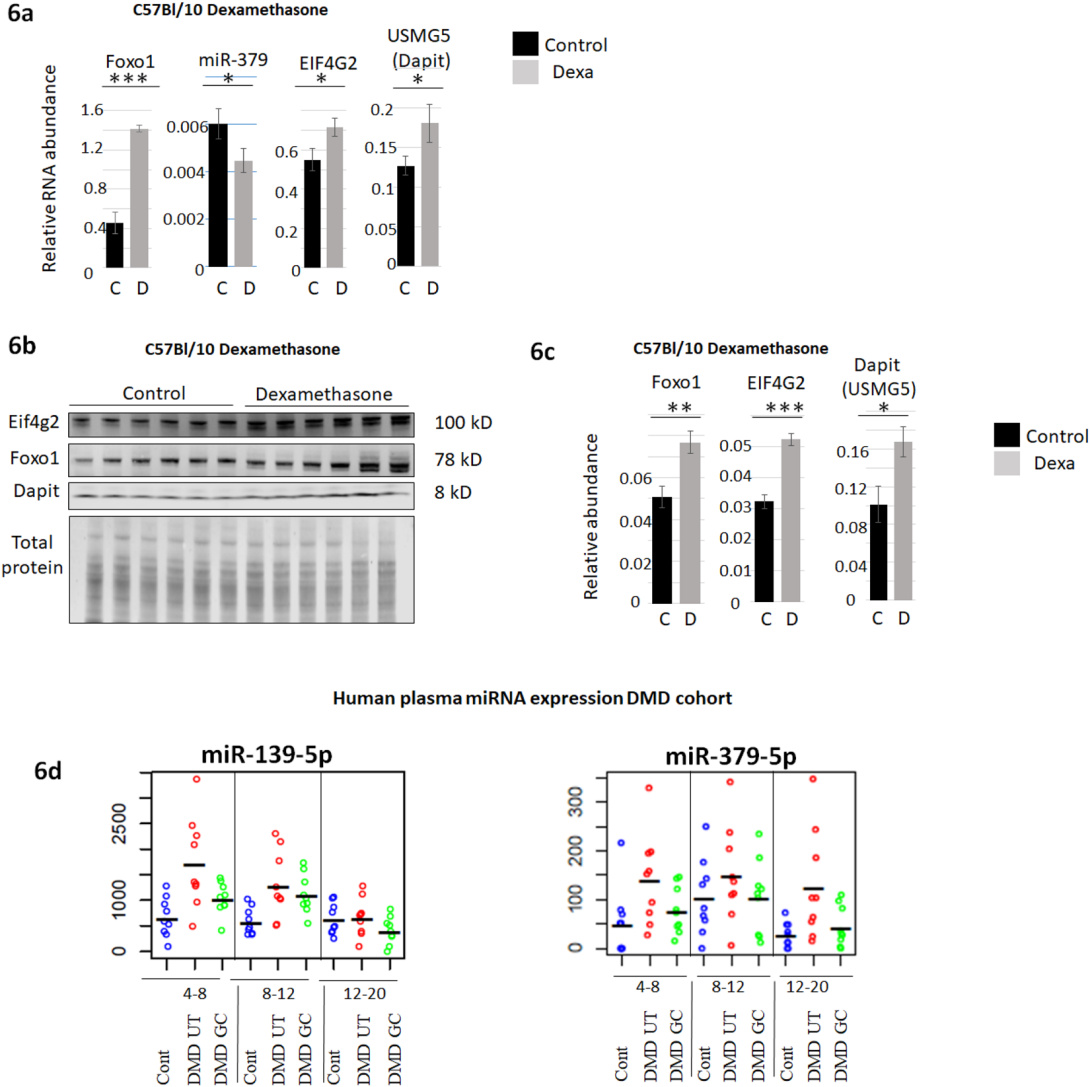
Glucocorticoid response of miR-379, EIF4G2 and DAPIT. (**a**) Foxo1, EIF4G2 and USMG5 mRNAs (normalized to p0 (*RPLP0*)), and miR-379-5p (normalized to U6/miR-93/miR-16), relative abundance in the gastrocnemius muscle of C57Bl/10 (n = 11), treated by dexamethasone (D), and untreated control (C). (**b**) and **(c**), Protein expression levels of Foxo1, EIF4G2 and DAPIT (normalized to total protein) in the gastrocnemius muscle of C57Bl/10 treated by dexamethasone (D) and untreated (C). (**d**) miR-139-5p and miR-379-5p expression in the plasma in a human cohort (each dot represents one patient, n = 9). Healthy control (Cont), DMD patients, untreated (DMD UT), and treated by glucocorticoid (DMD GC), of the age groups 4–8, 8–12 and 12–20 years old.

Because miR-379 was previously demonstrated to be glucocorticoid-responsive^28^, and considering the effect of GC on the phenotype of DMD, we investigated whether GC can have an effect on the muscle expression of miR-379, EIF4G2 and DAPIT. For this purpose, we treated mice with a subcutaneous injection of dexamethasone (2 mg /kg, n = 11) for 5 days. The upregulation of Foxo1 indicated the expected GC response. The expression of miR-379 was significantly reduced, while the mRNA levels EIF4G2 and DAPIT were significantly increased (Fig. 6a). In agreement, the protein levels of Foxo1, EIF4G2 and DAPIT were significantly upregulated (Fig. 6b,c). Lastly, we evaluated the GC response of miR-139 and miR-379 in DMD patients. In the absence of adequate muscle biopsies, we turned our attention to the circulating miRNAs. Blood plasma samples of 54 DMD and 27 healthy control patients were divided into three age groups. Of the DMD patients, half were treated by glucocorticoid. Both miR-139 and miR-379 were found upregulated in DMD plasma and partially normalized by GC treatment (FC = 0.625, p < 0.047 and FC = 0.710, p < 0.041, respectively for miR-139 and miR-379) **(**Fig. 6d**)**. Thus, in the mouse, GC treatment correlated with the reduced expression of miR-379 and with the increased levels of EIF4G2 and DAPIT in the muscle. In DMD patients, GC treatment correlated with normalized expression of miR-139 and 379 in the blood plasma.

## Discussion

Following the identification of a large number of dysregulated miRNAs in plasma samples from DMD patients and GRMD dogs, we have now demonstrated the dysregulation of many of these miRNAs, including Dlk1-Dio3 miRNAs, within the skeletal muscle, using the GRMD dog as a model of DMD. Furthermore, we have shown that the Dlk1-Dio3 miRNAs are not only dysregulated, but are in fact among the most highly dysregulated miRNAs in the GRMD dystrophic muscle, to a higher level than the myomiRs. This observation suggests an as yet unrecognized significant participation of these miRNAs in the dystrophic process. Of these Dlk1-Dio3 miRNAs, we selected miR-379 for further investigation. We showed subsequently that this miR is expressed within the myogenic lineage. Further investigation demonstrated an axis where miR-379 controls the expression of the EIF4G2 translation factor, which itself controls the translation of the mitochondrial protein DAPIT. We then demonstrated that this signaling pathway is not only dysregulated in DMD but also reversed toward normalization by a GC treatment. Importantly, we found that miR-379 and miR-139 are GC responsive following the determination of expression level in plasma samples of DMD patients. Thus, the data suggesting that miR-379 may participate in the GC response in the dystrophic muscle, linking therefore a glucocorticoid effect to DAPIT-related mitochondrial dysfunction and highlighting a new aspect of the DMD pathophysiology.

### The involvement of Dlk1-Dio3 miRNAs in muscle pathophysiology

The Dlk1-Dio3 miRNAs are processed from long noncoding RNA (lncRNA) genes expressed from the maternal chromosome of the highly conserved Dlk1-Dio3 imprinted genomic locus. A critical involvement of these lncRNAs in mammalian striated muscle development was demonstrated in mice, where the deletion of the Gtl2 lncRNA resulted in skeletal muscle developmental defect and perinatal death^48^. Another study demonstrated that the Dlk1-Dio3 lncRNAs and the processed miRNAs are upregulated in the myogenic lineage by the MEF2A transcription factor and that this pathway is involved in muscle differentiation and regeneration^49^. In a third example, Gao *et al*.^50^ created a mouse model with deletion of the miR-379-544 cluster that presented skeletal muscle hypertrophy. Two recent investigations, focused on muscle precursor cells, identified a large number of Dlk1-Dio3 miRNAs which are highly expressed in quiescent muscle satellite cells^37^, and participate in the control of their metabolic maturation and mitochondrial functions^51^. Taken together, these studies provide compelling evidence for the critical involvement of the Dlk1-Dio3 clustered miRNAs in muscle function. Relatively few studies have focused on the muscle functions of individual miRNAs of this locus. Of these, miR-431 has been proposed as promoting the regeneration of old and of dystrophic muscles, due to the inhibition respectively of SMAD4^52^, and Pax7^53^. miR-410 has been proposed as a participant in diverse cardiomyopathies^54^. As for miR-379, apart from the observations of its dysregulation in the serum and muscles in muscular dystrophies^13,20^, no previous publications have identified a role for this microRNA in muscle pathology.

### Dysregulation of the miR-379 EIF4G2 DAPIT mitochondria pathway in DMD disease

**Figure 7:**
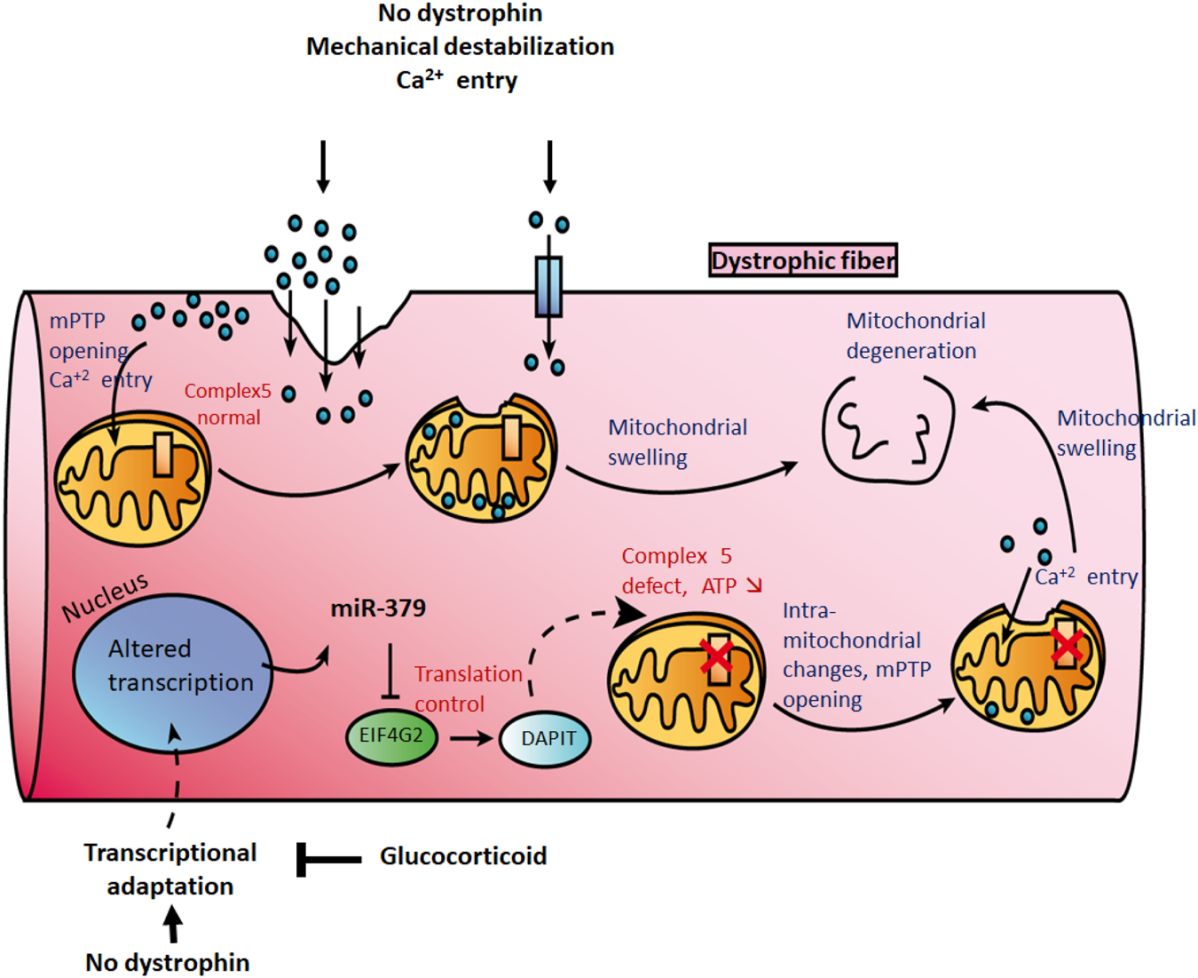
Revised model for mitochondria dysfunction in the DMD disease. Graphical model for mechanisms of mitochondrial dysfunction in the DMD disease. The Ca^2+^ hypothesis, which is presented on the model upper part, was proposed initially by Wrogemann and Pena at 1976^75^. Accordingly, in the absence of dystrophin, Ca^2+^ entry via sarcolemma leakiness and/or activated ion channels is initiating a cytoplasmic pathological cascade (see main text for details) that culminate by the opening of the mitochondrial permeability transition pore (mPTP) and mitochondrial degeneration. In the modified model, presented on the image lower part, it is proposed that the absence of dystrophin induces gene-expression changes, activating miR-379 expression, reducing EIF4G2 and DAPIT expression, reducing ATP synthase activity and ATP production, (see main text for details), which might synergizes with the external Ca^2+^ overload, to produce mitochondrial damage.

The mitochondrial Oxphos respiration, which is required for myoblast differentiation^55^, is dysfunctional in the muscles in DMD^8^, which suggested the possible involvement of EIF4G2 in muscular dystrophy. Of the potential mitochondrial targets of EIF4G2, we decided to study DAPIT, because it is associated to the ATP synthase complex and has been reported to be expressed higher in relatively moderate as compared severe DMD patients^24^. DAPIT is a mitochondrial ATP synthase non-catalytic subunit^38,39^, which is required for the dimerization of the ATP Synthase^40^. Thus, whereas DAPIT has no role in the production of ATP *per se*, the dimerization of mitochondrial ATP synthase is required for the shaping of the mitochondrial cristae^43,56–58^ and the subsequent optimal ATP production^25,59^. In agreement, it was shown that knocking down DAPIT in Hela cells resulted in approximately 40% reduced ATP production^42^. It is of particular interest that most recently a mutation in the DAPIT gene was found in Leigh syndrome, characterized by a severe neurodevelopmental regression and early childhood death, and at the cellular level by disorganized skeletal muscle mitochondria, reduced crista density and ATP production^25,43^. Important in the context of DMD disease, a 2011 paper identified higher DAPIT expression in muscle biopsies of DMD patients with a late, as compared to early, loss of walking capacity^24^. Taken all together, it is reasonable to hypothesize that reduced DAPIT expression may participate in mitochondrial dysfunction in the dystrophic muscle. Our data support the hypothesis that one cause of the mitochondrial defect in DMD is the upregulation of miR-379. Accordingly, in addition to mechanical destabilization and elevated cytosolic Ca^2+^, an increased expression of miR-379, results in reduced expression of EIF4G2, reduced expression of DAPIT, reduced ATP synthase dimerization and reduced ATP production. Thus, in agreement with a previous proposition^11^, our data supports a revised mitochondrial dysfunction model in DMD, in which a mitochondrial internal defect in DAPIT expression and ATP production may precede and synergize with an extra-mitochondrial damage cascade (Fig. 7**)**.

### The effect of glucocorticoid treatment in DMD

The mechanism by which the GCs protect the dystrophic muscle in DMD patients is yet not completely understood. It has been suggested that immunosuppressive and anti-inflammatory activities may mediate the beneficial GC effects. Additionally, it was shown recently that GCs are involved in the regulation of metabolic pathways^60^ and accelerated sarcolemma repair^61^. Here, we are proposing a link between GCs and the modulation of a critical mitochondrial function. Accordingly, the GC treatment would reduce the expression of both miR-379 and miR-139, and ultimately provide a positive effect on the mitochondrial function in DMD through the cascade identified here. A previous investigation has identified the upregulation of miR-379 in the liver of a mouse model for hyperglucocorticoidemia^28^. It is however well documented that the glucocorticoid transcription response can vary for a given target between up and down regulation in a cell type and physiological context dependent manner^62,63^. Of interest, a recent paper suggested that in tuberculosis patients, dexamethasone treatment provides protection not by immunosuppression (as previously proposed), but rather by reversing a signaling pathway that involves mitochondrial ATP depletion^64^, providing thus another example for GC reversion of mitochondrial dysfunction.

In summary, in the present study we identified a signaling pathway, which (1) links miR-379 to mitochondrial function, (2) is dysregulated in the dystrophic muscle, and (3) partially normalized by glucocorticoids. These findings supporting an updated model of mitochondrial dysfunction and glucocorticoid effect in DMD.

## Materials and Methods

### Animal care and use

All animals were handled according to French and European guidelines for human care and use of experimental animals. Procedures on GRMD dogs were approved by the ethical Committee Afssa/EnvA/UPEC n°CNRE-EA-16f under the number n° 12-095 / notice n° 20/12/12-19. Experiments on mice were approved by the ethical committee n°C2AE-51 of Evry and the regulatory affairs of the French Ministry of Research (MESRI) under the number APAFIS#3519. Dogs were bred and housed at the facility of the National Veterinary School of Alfort (France). Canine biopsies from the *Biceps femoris* and *Sartorius cranialis* were sampled at the age of 2 months on normal (n = 9), severely (n = 10) and moderately (n = 9) affected dogs. GRMD clinical stratification was based on the ambulatory status of the dogs at the age of 6 months: severe cases being characterized by a loss of ambulation before this age, and moderately affected dogs, still ambulant at the age of 6 months and usually keeping the ambulation ability^65^.

C57Bl10 (WT) and *Mdx (*C57BL/10ScSn-*Dmdmdx*/J) mice were obtained from Charles River laboratories. Mice were housed in a SPF barrier facility with 12-h light, 12-h dark cycles, and were provided with food and water *ad libitum*.

### *In vivo* mice experiments

Subcutaneous injections of 2 mg/kg/dose of dexamethasone (Sigma Aldrich, D2915) were performed over 5 consecutive days at 7–8 weeks of age in C57Bl/10 (n = 11) and mdx (n = 6). Mice were sacrificed the day after the last injection and skeletal muscles were frozen.

### RNA Expression analysis in the dog study

Total RNA was extracted from frozen muscles or cells using Trizol (Thermo Fisher Scientific, Waltham, MA, USA), according to the manufacturer protocol. Total RNA was quantified using a Nanodrop 8000 spectrophotometer (Labtech, Wilmington Delaware). To measure microRNA expression, 10 ng of total RNA were reverse-transcribed using Exiqon Universal cDNA Synthesis Kit II (Exiqon). Real-time PCR was performed using LightCycler 480 system (Roche, Bâle, Switzerland), with the Exiqon miRNAs assays and miRCURY LNA SYBR Green Master Mix (Exiqon) for the 34 miRNAs indicated in Table 1 and three normalizers. Results obtained with hsa-miR-93-5p, hsa-miR-16-5p and the U6 small nuclear RNA were used to normalize data across samples. Each experiment was performed in duplicate. To measure gene expression, 1 µg of total RNA was reverse-transcribed using a mixture of random oligonucleotides and oligo-dT and RevertAid H Minus First Strand cDNA Synthesis Kit (Thermo Fisher Scientific). Real-time PCR was performed using LightCycler 480 system (Roche, Bâle, Switzerland) with the following Taqman Gene Expression assays (Thermo Fisher Scientific): Col6A3 (Cf02702942_m1), *Cd11b* (Cf02663762_m1), *Myh8* (Cf02730386_m1), EIF4G2 (Hs00154952_m1), PDK1 (Hs01561847_m1), UQCR10 (Hs00912476_m1), USMG5/DAPIT (Hs00910071_g1), Myogenin (Hs01072232_m1) and MYOD1 (Hs00159528_m1) according to the manufacturer’s protocol. Results obtained with the ubiquitous acidic ribosomal phosphoprotein gene (*P0/ RPLP0*) were used to normalize the data across samples. The primers and Taqman probe used for *P0* amplification were: P0.F (5′-CTCCAAGCAGATGCAGCAGA-3′), P0.R (5′-ACCATGATGCGCAAGGCCAT-3′) and P0.P (5′-CCGTGGTGCTGATGGGCAAGAA-3′). Amplification of human Pax3 and Myf5 was performed using primers AGTTCCATCAGCCGCATC and TTCTTCTCGCTTTCCTCTGC for Pax3, AACCCTCAAGAGGTGTACCAC and AGGACTGTTACATTCGGGCAT for Myf5.

### Satellite cell isolation from mouse muscles

Satellite cells were isolated from limb muscles as follows. All hind limb muscles of 5-weeks old mice were collected and minced together in a Petri dish using scissors. The samples were put in a digestion medium [DMEM, Dispase II (3U/ml), Collagenase A (0.5 U/ml), 0.2% BSA, Pen-Strep; 10 ml for one mouse] for two hours at 37 °C with gentle shaking. Mononucleated cells were collected after passing through successive strainers (mesh diameters of 100, 70 and 40 µm) in ice-cold DMEM to eliminate fibers and debris. After washing in ice-cold PBS 0.1% BSA and centrifugation at 2000 rpm for 2 min at 4 °C, the samples were treated using 1 ml Red Blood Cell Lysis (Versalyse – A09777 Beckman Coulter) for 5–10 min at RT to eliminate the blood cells. After a final centrifugation at 2000 rpm for 2 min at 4 °C, the cells were resuspended in 1 ml ice-cold PBS 0.1% BSA before FACS sorting for CD45- (BD Biosciences 559864), Sca1- (BD Biosciences 553108), CD31-(BD Biosciences 551262), and Vcam1 + (BD Biosciences 553331) cells.

### Cell culture and differentiation

Myogenic cell line (AB1190, paravertebral muscle, 16 years old healthy male)^35^, were grown in proliferation medium (Promocell, Ref. C-23060) supplemented with 15% of fetal calf serum and 1% GlutaMAX (Thermo Scientific, Ref. 35050061). Differentiation into myotubes was performed at 80% confluence using differentiation medium (Promocell, Ref. C-23061) supplemented with the according Supplemental Mix provided by the manufacturer. Differentiated myotubes were blindly counted (n = 3, average myotubes for 5 images per transfection) on phase microscopy images. The M180 human iPS cell line, from a healthy donor (14-year-old female)^47^, were grown in mTESR1 medium (Stemcell, Ref. 85850) supplemented by Rock Inhibitor (Stemcell, Ref.10,72304 µM final and using Matrigel as coating (Corning, Ref. 354234).

M180 iPS cells were differentiated toward skeletal muscle lineage (skMPC) using commercial media designed from Caron’s work^66^. (Skeletal Muscle Induction medium SKM01, Myoblast Cell Culture Medium SKM02, Myotube Cell Culture Medium SKM03, (AMSbio, Milton Park, Abingdon, OX14 4SE, UK)). This protocol is a 2D directed differentiation that uses 3 consecutive defined media (SKM01 from day 0 to 10, SKM02 from day 10 to 17 and SKM03 from day 17 to d25) and cell passages at day 7 and day 10. Cells were seeded at 3,500 cells/cm^2^ at day 0, 13,000 cells/cm^2^ at day 7 and 5000 cells/cm^2^ at day 10 on BioCoat Collagen I cultureware (356485, Corning Incorporated). Skeletal Muscle Precursor Cells M180 were frozen at day 17 of differentiation until further use.

### Transfection

For miR-379-5p overexpression, immortalized myoblasts were plated (80 000 cells) in 6-well plates. The day after the cells were transfected by hsa-miR-379-5p mimics (miRCURY LNA, Exiqon, Ref. 470847-001) using Lipofectamine RNAiMAX (Thermo Scientific, Ref. 13778075) at 0.5, 1 or 5 nM final concentrations, for 48 hours until analysis. A scramble LNA was used as negative control (Negative Control 5, miRCURY LNA, Exiqon, Ref. 479904-001). For gene inhibition experiments, cells were transfected by MISSIONesiRNA for human EIF4G2 and USMG5 (DAPIT) (Sigma Aldrich, Ref. EHU032391-50UG and EHU103831-50UG, respectively) at 10, 15 or 30 nM, using Lipofectamine RNAiMAX (Thermofisher 13778150). The negative control was the universal-1 negative control (MISSION = , Sigma Aldrich, Ref. SIC001-5×1NMOL). For the transfection of *in vitro* differentiated myotubes (for the ATP/ADP quantification experiment), five-day *in vitro* differentiated AB1190 myotubes were transfected (Lipofectamine RNAiMAX Thermofisher 13778150) with either human Si-USMG5 (Dapit) or Si-scramble, at a final concentration 30 nM, for 48 hours. Dapit knockdown (60%) was confirmed by RT-qPCR.

### Histological staining and labelling of muscle sections

Mouse muscles (*psoas*, *tibialis anterior*, *quadriceps, gastrocnemius* and *diaphragm*) were sampled and frozen in isopentane cooled in liquid nitrogen. Transverse cryosections (8–10 µm) were prepared from frozen muscles and were processed for hematoxylin-phloxine-saffron (HPS) histological staining.

### Immunostaining of single myofibers

Gastrocnemius muscles from 10-week old C57Bl/10 mice were dissected from tendon to tendon. The muscle was washed twice in PBS and incubated in 1% w/v Collagenase A (Sigma, 11088793001)/DMEM for 90 minutes on an orbital shaker at 25 rpm, in 37 °C incubator. Single myofibers were released, washed in PBS, fixed immediately in 3.7% PFA for immuno-histofluorescence staining. Myofibers were permeabilised for 15 minutes in 0.3% Triton X-100 (Sigma Aldrich) and blocked in Blocking Buffer, of 5% Goat Serum (Gibco) + 10% Fetal Bovine Serum in 1X PBS, for 1 hour at room temperature. Fibers were then incubated with primary antibodies diluted in 1/10 Blocking Buffer (Usmg5, ProteIntech 17716-1-AP, 1:100; ATP5a, Abcam sc-58613, 1:100) overnight at 4 °C. Samples were washed with 1X PBS twice and incubated with Alexa-conjugated secondary antibodies diluted in 1/10 Blocking Buffer (Life Technologies, 1/1000) for 45 min at room temperature. After washing twice in 1X PBS, samples were mounted in DAPI Fluoromount-G (Southern Biotech, O100-20). Confocal microscopy images were acquired with Leica TCS-SP8, using a 63x oil objective.

### Mitochondrial activity measurement

The measure of activity of complexes I and V of the mitochondria respiratory chain was performed by spectrophotometry using a Cary 60 apparatus (Agilent Technologies), following a published protocol^67^. Each sample contained approximatively 2 million cells. For normalization, the level of protein was measured by BCA Pierce Assay (Thermo Scientific) at the end of the experiment. Complex activities were calculated according to the Beer-Lambert formula, then a ratio between the 2 activities was calculated.

### ATP/ADP of *in vitro* differentiated myotubes

ATP/ADP ratio was assayed with the ATP/ADP assay kit of Sigma (MAK135) following manufacturer’s instructions. AB1190 myoblast were grown in a low glucose (1 g/L) DMDM (Thermo-Fisher, 22320-022), 15% fetal calf serum. Confluent myoblasts were shifted to a low glucose/low horse-serum (5%), DMEM differentiation medium, for 5 days until the appearance of myotubes. *In vitro* differentiated myotubes were transfected (as described in the transfections section). ATP/ADP ratio was assayed with the ATP/ADP assay kit of Sigma (MAK135) following manufacturer’s instructions.

### Protein analysis

Proteins were extracted from tissues or cells by cell lysis buffer (RIPA Buffer, Thermo Scientific), Protease Inhibitor Cocktails (Complete PIC, Roche) and benzonase 1:10 000 (Millipore) after homogenization using pellet pestles in the case of tissues. The samples were prepared following the NuPAGE Gel protocol (ThermoFisher). The proteins were separated using a NuPAGE pre-cast 4–12% Bis-Tris gel and then transferred to nitrocellulose membrane with iBlot2 Dry Blotting system (ThermoFisher) using the 8 min30/20 V program in the case of detection of proteins with a size higher than 10 kDA. In the case of proteins with lower size, the proteins were separated using a NuPAGE pre-cast 10–20% Tricine gel and then transferred to PVDF membrane with iBlot2 Dry Blotting system using the 7 minutes/10 V program. Detection of proteins was performed using standard Odyssey protocol by incubation with primary specific antibodies: EIF4G2 (BD Biosciences, 610742), PDK1 (Santa Cruz, sc-293160), USMG5/DAPIT (Abcam, ab108225), Actin (Sigma Aldrich, A2066) and α-actinin (Santa Cruz, sc-15335). All antibodies were diluted between 1:2000 and 1:1000 in PBS/Odyssey 1:1 buffer. Membranes were incubated with antibodies either 2 h RT or 4 °C overnight. Western blot were revealed using Odyssey secondary antibodies donkey anti mouse (DAM) 680, donkey anti goat (DAG) 680 and donkey anti rabbit (DAR) 800 diluted 1:1000, 1H30 hour at room temperature and then scanned with the Odyssey machine. Band density was quantified using the Image Studio Lite 4.0 software (LI-COR Biosciences, Lincoln, NE). The integrated density of assayed proteins were normalized by the Alpha-actinin or alpha actin values.

### Ago 1/2/3 immunoprecipitation

The Ago 1/2/3 immunoprecipitation was performed using the miRNA Target IP (Active Motif, n°25500) following the manufacturer’s protocol. After immunoprecipitation, RT-qPCR was performed with Taqman Gene Expression primers of target genes of interest (PDK1, EIF4G2 and UQCR10) with P0 as a normalizer. The IP-Ago/IP-Negative control ratio was calculated for each sample, and this ratio was compared between hsa-miR-379-5p-treated samples and scramble-treated samples. A ratio higher in hsa-miR-379-5p-treated samples for an mRNA was considered positive enrichment of the IP-Ago fraction. A ratio at least as high as the positive control one indicated that the mRNA was a target of miR-379-5p.

### Measure of miRNA level in human plasma

The human study (DMD patients and controls) was conducted according the principles of the declaration of Helsinki “ethical principles for medical research”, and was specifically approved by the ethical committee CPP Ile de France VI, on July 20, 2010, and the Comité d’Ethique (412) du CHR La Citadelle (Liège, Belgium) January 26, 2011. Samples were collected from individuals under a written informed consent of parents or legal guardians. DMD patients and healthy controls were admitted from 10 European medical centers, two of them from Belgium, one from Romania, and seven from France. The entire studied miRNA-sequencing cohort was composed of 54 DMD patients and 27 healthy controls (N = 81) divided into 9 groups of 9 subjects, composed of three age groups (4–8, 8–12, and 12–20 years old), of DMD patients treated by GCs, of untreated DMD patients, and of age matching healthy controls. In the younger age group (4–8 years old), the same donors contributed to the GC-treated and untreated samples, with untreated samples obtained before and treated samples after their first GC treatment, with less than 6-month interval. Peripheral blood samples were collected into 5 ml K3EDTA tubes (Greiner Bio-One). Plasma was separated from buffy coat and red blood cells after 10 minutes centrifugation at 1800 g and stored at −80 °C. Three hundred µl human plasma were used for RNA extractions, employing the mirVana PARIS kit (ThermoFisher). MiRNA sequencing was performed by Integragen (Evry, France). Library cloning was modified from^68^. Libraries were quantified by qPCR, to load precisely 7pM pool per line of HiSeq Flow-Cell. The HiSeq. 36b and index (barcode) sequencing was done as instructed (Illumina) with a SBS V3 kit leading on 150 million passing filter clones. All clean reads were compared to the Rfam database (http://rfam.xfam.org/), repeatmasker (http://www.repeatmasker.org/, UCSC download 01/04/2014), and the NCBI RefSeq.^69^, download 10/04/2014). Unique miR reads and their copy numbers were aligned with miRanalyzer online software^70^, using Ensembl human gene browser (genome assembly GRCh38) and (miRbase v20, June 2013^71^). MiR count Raw data were processed for differential expression analysis with the Deseq. 2 R and ggplot2 packages^72^. The significance of miRNAs differential expression was ranked by T-test, with a false discovery rate (FDR) correction according^73^. P values and fold change values were used for the classification of differential expression.

### Statistical analysis

Expression results were processed using R version 3.6.0 (2019-04-26) and PCA analysis using the FactoMineR packages^74^. Data for each group are represented as the means plus standard error of the mean (SEM). P-values were calculated by the Student test. Statistical significance is represented by stars: (*) for P < 0.05, (**) for P < 0.01 (***), for P < 0.001 and (****) for P < 0.0001.

### Supplementary information

Supplementary Information.

## References

[1] Mah JK (2014). A systematic review and meta-analysis on the epidemiology of Duchenne and Becker muscular dystrophy. Neuromuscul. Disord..

[2] Passamano L (2012). Improvement of survival in Duchenne Muscular Dystrophy: retrospective analysis of 835 patients. Acta Myol..

[3] Kieny P (2013). Evolution of life expectancy of patients with Duchenne muscular dystrophy at AFM Yolaine de Kepper centre between 1981 and 2011. Ann. Phys. Rehabil. Med..

[4] Birnkrant DJ (2018). Diagnosis and management of Duchenne muscular dystrophy, part 1: diagnosis, and neuromuscular, rehabilitation, endocrine, and gastrointestinal and nutritional management. Lancet Neurol..

[5] Gloss D, Moxley RT, Ashwal S, Oskoui M (2016). Practice guideline update summary: Corticosteroid treatment of Duchenne muscular dystrophy. Neurology.

[6] McDonald CM (2018). Long-term effects of glucocorticoids on function, quality of life, and survival in patients with Duchenne muscular dystrophy: a prospective cohort study. Lancet.

[7] Bello L (2015). Prednisone/prednisolone and deflazacort regimens in the CINRG Duchenne Natural History Study. Neurology.

[8] Timpani CA, Hayes A, Rybalka E (2015). Revisiting the dystrophin-ATP connection: How half a century of research still implicates mitochondrial dysfunction in Duchenne Muscular Dystrophy aetiology. Med. Hypotheses.

[9] Allen DG, Whitehead NP, Froehner SC (2016). Absence of Dystrophin Disrupts Skeletal Muscle Signaling: Roles of Ca 2+, Reactive Oxygen Species, and Nitric Oxide in the Development of Muscular Dystrophy. Physiol. Rev..

[10] Burr AR, Molkentin JD (2015). Genetic evidence in the mouse solidifies the calcium hypothesis of myofiber death in muscular dystrophy. Cell Death Differ..

[11] Kelly-Worden M, Thomas E (2014). Mitochondrial Dysfunction in Duchenne Muscular Dystrophy. Open. J. Endocr. Metab. Dis..

[12] Rybalka Emma, Timpani Cara A., Cooke Matthew B., Williams Andrew D., Hayes Alan (2014). Defects in Mitochondrial ATP Synthesis in Dystrophin-Deficient Mdx Skeletal Muscles May Be Caused by Complex I Insufficiency. PLoS ONE.

[13] Eisenberg I (2007). Distinctive patterns of microRNA expression in primary muscular disorders. Proc Natl Acad Sci USA.

[14] Vignier N (2013). Distinctive serum miRNA profile in mouse models of striated muscular pathologies. PLoS One.

[15] Matsuzaka Y (2014). Three novel serum biomarkers, miR-1, miR-133a, and miR-206 for Limb-girdle muscular dystrophy, Facioscapulohumeral muscular dystrophy, and Becker muscular dystrophy. Environ. Health Prev. Med..

[16] Zaharieva IT (2013). Dystromirs as serum biomarkers for monitoring the disease severity in duchenne muscular dystrophy. PLoS One.

[17] Roberts TC (2012). Expression Analysis in Multiple Muscle Groups and Serum Reveals Complexity in the MicroRNA Transcriptome of the mdx Mouse with Implications for Therapy. Mol Ther Nucleic Acids.

[18] Mizuno H (2011). Identification of muscle-specific microRNAs in serum of muscular dystrophy animal models: promising novel blood-based markers for muscular dystrophy. PLoS One.

[19] Cacchiarelli D (2011). miRNAs as serum biomarkers for Duchenne muscular dystrophy. EMBO Mol Med.

[20] Jeanson-Leh L (2014). Serum Profiling Identifies Novel Muscle miRNA and Cardiomyopathy-Related miRNA Biomarkers in Golden Retriever Muscular Dystrophy Dogs and Duchenne Muscular Dystrophy Patients. Am. J. Pathol..

[21] Li X (2014). Circulating Muscle-specific miRNAs in Duchenne Muscular Dystrophy Patients. Mol. Ther. Nucleic Acids.

[22] Hu J (2014). Serum miR-206 and other muscle-specific microRNAs as non-invasive biomarkers for Duchenne muscular dystrophy. J. Neurochem..

[23] Kornegay Joe N., Bogan Janet R., Bogan Daniel J., Childers Martin K., Li Juan, Nghiem Peter, Detwiler David A., Larsen C. Aaron, Grange Robert W., Bhavaraju-Sanka Ratna K., Tou Sandra, Keene Bruce P., Howard James F., Wang Jiahui, Fan Zheng, Schatzberg Scott J., Styner Martin A., Flanigan Kevin M., Xiao Xiao, Hoffman Eric P. (2012). Canine models of Duchenne muscular dystrophy and their use in therapeutic strategies. Mammalian Genome.

[24] Pegoraro E (2011). SPP1 genotype is a determinant of disease severity in Duchenne muscular dystrophy. Neurology.

[25] Barca E (2018). USMG5 Ashkenazi Jewish founder mutation impairs mitochondrial complex V dimerization and ATP synthesis. Hum. Mol. Genet..

[26] Israeli D (2016). Circulating miRNAs are generic and versatile therapeutic monitoring biomarkers in muscular dystrophies. Sci. Rep..

[27] McGreevy JW, Hakim CH, McIntosh MA, Duan D (2015). Animal models of Duchenne muscular dystrophy: from basic mechanisms to gene therapy. Dis. Model. Mech..

[28] de Guia RM (2015). microRNA-379 couples glucocorticoid hormones to dysfunctional lipid homeostasis. EMBO J..

[29] Arnold CP (2011). MicroRNA programs in normal and aberrant stem and progenitor cells. Genome Res..

[30] Vlachos IS (2015). DIANA-miRPath v3.0: deciphering microRNA function with experimental support. Nucleic Acids Res..

[31] Agarwal, V., Bell, G. W., Nam, J.-W. & Bartel, D. P. Predicting effective microRNA target sites in mammalian mRNAs. Elife 4, (2015). 10.7554/eLife.05005 PMC4532895 26267216

[32] Liberman N, Marash L, Kimchi A (2009). The translation initiation factor DAP5 is a regulator of cell survival during mitosis. Cell Cycle.

[33] Yamanaka S (2000). Essential role of NAT1/p97/DAP5 in embryonic differentiation and the retinoic acid pathway. EMBO J..

[34] Yoffe Y (2016). Cap-independent translation by DAP5 controls cell fate decisions in human embryonic stem cells. Genes Dev..

[35] Mamchaoui K (2011). Immortalized pathological human myoblasts: towards a universal tool for the study of neuromuscular disorders. Skelet. Muscle.

[36] Li Z, Shen J, Chan MTV, Wu WKK (2017). MicroRNA-379 suppresses osteosarcoma progression by targeting PDK1. J. Cell. Mol. Med..

[37] Castel D (2018). Small-RNA sequencing identifies dynamic microRNA deregulation during skeletal muscle lineage progression. Sci. Rep..

[38] Meyer B, Wittig I, Trifilieff E, Karas M, Schägger H (2007). Identification of Two Proteins Associated with Mammalian ATP Synthase. Mol. Cell. Proteomics.

[39] Chen R, Runswick MJ, Carroll J, Fearnley IM, Walker JE (2007). Association of two proteolipids of unknown function with ATP synthase from bovine heart mitochondria. FEBS Lett..

[40] He J (2018). Assembly of the membrane domain of ATP synthase in human mitochondria. Proc. Natl. Acad. Sci..

[41] Kontro H, Hulmi JJ, Rahkila P, Kainulainen H (2012). Cellular and tissue expression of DAPIT, a phylogenetically conserved peptide. Eur. J. Histochem..

[42] Ohsakaya, S., Fujikawa, M., Hisabori, T. & Yoshida, M. Knockdown of DAPIT (diabetes-associated protein in insulin-sensitive tissue) results in loss of ATP synthase in mitochondria. **286**, (2011). 10.1074/jbc.M110.198523 PMC3121504 21345788

[43] Siegmund SE (2018). Three-Dimensional Analysis of Mitochondrial Crista Ultrastructure in a Patient with Leigh Syndrome by *In Situ* Cryoelectron Tomography. iScience.

[44] Diaz-Ruiz R, Uribe-Carvajal S, Devin A, Rigoulet M (2009). Tumor cell energy metabolism and its common features with yeast metabolism. Biochim. Biophys. Acta - Rev. Cancer.

[45] Lisowski P, Kannan P, Mlody B, Prigione A (2018). Mitochondria and the dynamic control of stem cell homeostasis. EMBO Rep..

[46] Facucho-Oliveira JM, St. John JC (2009). The Relationship Between Pluripotency and Mitochondrial DNA Proliferation During Early Embryo Development and Embryonic Stem Cell Differentiation. Stem Cell Rev. Reports.

[47] Massouridès E (2015). Dp412e: a novel human embryonic dystrophin isoform induced by BMP4 in early differentiated cells. Skelet. Muscle.

[48] Zhou Y (2010). Activation of paternally expressed genes and perinatal death caused by deletion of the Gtl2 gene. Development.

[49] Snyder CM (2013). MEF2A regulates the Gtl2-Dio3 microRNA mega-cluster to modulate WNT signaling in skeletal muscle regeneration. Development.

[50] Gao Y (2014). The H19/let-7 double-negative feedback loop contributes to glucose metabolism in muscle cells. Nucleic Acids Res..

[51] Wüst S (2018). Metabolic Maturation during Muscle Stem Cell Differentiation Is Achieved by miR-1/133a -Mediated Inhibition of the Dlk1-Dio3 Mega Gene Cluster. Cell Metab..

[52] Lee K-P (2015). miR-431 promotes differentiation and regeneration of old skeletal muscle by targeting Smad4. Genes Dev..

[53] Wu R (2015). MicroRNA-431 accelerates muscle regeneration and ameliorates muscular dystrophy by targeting Pax7 in mice. Nat. Commun..

[54] Clark AL (2016). miR-410 and miR-495 Are Dynamically Regulated in Diverse Cardiomyopathies and Their Inhibition Attenuates Pathological Hypertrophy. PLoS One.

[55] Sin J (2016). Mitophagy is required for mitochondrial biogenesis and myogenic differentiation of C2C12 myoblasts. Autophagy.

[56] Paumard P (2002). The ATP synthase is involved in generating mitochondrial cristae morphology. EMBO J..

[57] Strauss M, Hofhaus G, Schröder RR, Kühlbrandt W (2008). Dimer ribbons of ATP synthase shape the inner mitochondrial membrane. EMBO J..

[58] Hahn A (2016). Structure of a Complete ATP Synthase Dimer Reveals the Molecular Basis of Inner Mitochondrial Membrane Morphology. Mol. Cell.

[59] Cogliati S (2013). Mitochondrial Cristae Shape Determines Respiratory Chain Supercomplexes Assembly and Respiratory Efficiency. Cell.

[60] Morrison-Nozik A (2015). Glucocorticoids enhance muscle endurance and ameliorate Duchenne muscular dystrophy through a defined metabolic program. Proc. Natl. Acad. Sci..

[61] Quattrocelli M (2017). Intermittent glucocorticoid steroid dosing enhances muscle repair without eliciting muscle atrophy. J. Clin. Invest..

[62] Weikum ER, Knuesel MT, Ortlund EA, Yamamoto KR (2017). Glucocorticoid receptor control of transcription: precision and plasticity via allostery. Nat. Rev. Mol. Cell Biol..

[63] Cohen DM, Steger DJ (2017). Nuclear Receptor Function through Genomics: Lessons from the Glucocorticoid Receptor. Trends Endocrinol. Metab..

[64] Gräb J (2019). Corticosteroids inhibit Mycobacterium tuberculosis-induced necrotic host cell death by abrogating mitochondrial membrane permeability transition. Nat. Commun..

[65] Barthélémy I (2014). Predictive markers of clinical outcome in the GRMD dog model of Duchenne muscular dystrophy. Dis. Model. Mech..

[66] Caron L (2016). A Human Pluripotent Stem Cell Model of Facioscapulohumeral Muscular Dystrophy-Affected Skeletal Muscles. Stem Cells Transl. Med..

[67] Bénit P (2006). Three spectrophotometric assays for the measurement of the five respiratory chain complexes in minuscule biological samples. Clin. Chim. Acta..

[68] Vigneault, F. *et al*. High-throughput multiplex sequencing of miRNA. Curr. Protoc. Hum. Genet. Chapter 11, Unit 11.12.1-10 (2012). 10.1002/0471142905.hg1112s73 PMC3673877 22470142

[69] Pruitt KD, Tatusova T, Brown GR, Maglott DR (2012). NCBI Reference Sequences (RefSeq): current status, new features and genome annotation policy. Nucleic Acids Res..

[70] Hackenberg M, Rodríguez-Ezpeleta N, Aransay AM, Rodriguez-Ezpeleta N, Aransay A (2011). M. miRanalyzer: an update on the detection and analysis of microRNAs in high-throughput sequencing experiments. Nucleic Acids Res..

[71] Kozomara A, Griffiths-Jones S (2014). miRBase: annotating high confidence microRNAs using deep sequencing data. Nucleic Acids Res..

[72] Anders S (2013). Count-based differential expression analysis of RNA sequencing data using R and Bioconductor. Nat. Protoc..

[73] Benjamini Y, Hochberg Y (1995). Controlling the False Discovery Rate: a Practical and Powerful Approach to Multiple Testing. J. R. Stat. Soc..

[74] Lê S, Josse J, Husson F (2008). FactoMineR: An R Package for Multivariate Analysis. J. Stat. Softw..

[75] Wrogemann K, Pena SD (1976). Mitochondrial calcium overload: A general mechanism for cell-necrosis in muscle diseases. Lancet (London, England).

