# Author Correction: miR-379 links glucocorticoid treatment with mitochondrial response in Duchenne muscular dystrophy

**DOI:** 10.1038/s41598-024-57483-3

**Published:** 2024-03-28

**Authors:** Mathilde Sanson, Ai Vu Hong, Emmanuelle Massourides, Nathalie Bourg, Laurence Suel, Fatima Amor, Guillaume Corre, Paule Bénit, Inès Barthelemy, Stephane Blot, Anne Bigot, Christian Pinset, Pierre Rustin, Laurent Servais, Thomas Voit, Isabelle Richard, David Israeli

**Affiliations:** 1 Généthon INSERM, UMR_S951, INTEGRARE research Unit, 91000 Evry, France; 2 ISTEM, Inserm UMR 861, Evry, France; 3 Inserm U955-E10, IMRB, Université Paris Est, Ecole nationale vétérinaire d’Alfort, 94700 Maisons-Alfort, France; 4 Center for Research in Myology UMRS974, Sorbonne Université, INSERM, Myology Institute, Paris, France; 5 INSERM, UMR S1141, Hôpital Robert Debré, Paris, France; 6 MDUK Oxford Neuromuscular Centre, Department of Paediatrics, University of Oxford, Oxford, UK; 7 Division of Child Neurology, Centre de Références des Maladies Neuromusculaires, Department of Pediatrics, University Hospital Liège & University of Liège, Liège, Belgium; 8 NIHR Great Ormond Street Hospital Biomedical Research Centre and Great Ormond Street Institute of Child Health, University College London, London, UK

Correction to: *Scientific Reports* 10.1038/s41598-020-66016-7, published online 04 June 2020

The original version of this Article contained errors in the names of the authors Mathilde Sanson, Ai Vu Hong, Emmanuelle Massourides, Nathalie Bourg, Laurence Suel, Fatima Amor, Guillaume Corre, Paule Bénit, Inès Barthelemy, Stephane Blot, Anne Bigot, Christian Pinset, Pierre Rustin, Laurent Servais, Thomas Voit, Isabelle Richard & David Israeli, which were incorrectly given as M. Sanson, A. Vu Hong, E. Massourides, N. Bourg, L. Suel, F. Amor, G. Corre, P. Bénit, I. Barthélémy, S. Blot, A. Bigot, C. Pinset, P. Rustin, L. Servais, T. Voit, I. Richard & D. Israeli.

The original Article and accompanying Supplementary Information file have been corrected.

