# Supplementary material for: miR-379 links glucocorticoid treatment with mitochondrial response in Duchenne muscular dystrophy
Source: Sci Rep. 2020 Jun 4;10:9139. doi: 10.1038/s41598-020-66016-7 (PMC7272451; doi:10.1038/s41598-020-66016-7)
Supplement: Supplementary file 1 — Supplementary Information. [file 41598_2020_66016_MOESM1_ESM.docx]

**miR-379 links glucocorticoid treatment with mitochondrial response in Duchenne muscular dystrophy**

Mathilde Sanson^1^, Ai Vu Hong^1^, Emmanuelle Massourides^2^, Nathalie Bourg^1^, Laurence Suel^1^, Fatima Amor^1^, Guillaume Corre^1^, Paule Bénit^5^, Inès Barthelemy^3^, Stephane Blot^3^, Anne Bigot^4^, Christian Pinset^2^, Pierre Rustin^5^, Laurent Servais^6,7^, Thomas Voit^8^, Isabelle Richard^1^, David Israeli^1^

*^1^ Généthon INSERM, U951, INTEGRARE research unit Evry, F-91002, France*

*^2^ ISTEM, Inserm UMR 861, Evry, France*

*^3^ Inserm U955-E10, IMRB, Université Paris Est, Ecole nationale vétérinaire d’Alfort, 94700, Maisons-Alfort, France*

*^4^ Center for Research in Myology UMRS974, Sorbonne Université, INSERM, Myology Institute, Paris, France*

*^5^ INSERM, UMR S1141, Hôpital Robert Debré, Paris, France*

*^6^ MDUK Oxford Neuromuscular Center, Department of Paediatrics, University of Oxford, UK*

*^7^ Division of Child Neurology, Centre de Références des Maladies Neuromusculaires, Department of Pediatrics, University Hospital Liège & University of Liège, Belgium*

*^8^ NIHR Great Ormond Street Hospital Biomedical Research Centre and Great Ormond Street Institute of Child Health, University College London, UK*

**Corresponding Author:**

**David Israeli**

Généthon, 1 rue de l’Internationale, 91000 Evry, France

Tel: +33-1 69 47 29 67

E-mail: israeli@genethon.fr

**Sanson et al, Supplemental table 1**

References for dysregulation of the miRNAs that were selected for the present study

|  | **miR-name** | ***Dysregulation in GRMD serum***  ***Jeanson Leh et al*** | **Dysregulation in mdx serum Vignier et al** | **Dysregulation in DMD cohort, in preparation** |
| --- | --- | --- | --- | --- |
| 1 | **miR-30e-3p** |  | Up | ✓ |
| 2 | **miR-30e-5p** |  |  | ✓ |
| 3 | **miR-128-3p** | Up |  | ✓ |
| 4 | **miR-149-5p** | Down | Up | ✓ |
| 5 | **let-7g-5p** |  | Down | ✓ |
| 6 | **miR-103a-3p** |  |  | ✓ |
| 7 | **miR-378a-5p** | Up | Up | ✓ |
| 8 | **miR-133b** | Up | Up | ✓ |
| 9 | **miR-206** | Up |  | ✓ |
| 10 | **miR-106b-3p** |  |  | ✓ |
| 11 | **miR-106b-5p** |  |  |  |
| 12 | **miR-320a** |  |  | ✓ |
| 13 | **let-7d-5p** |  |  | ✓ |
| 14 | **miR-1307-3p** |  |  | ✓ |
| 15 | **miR-139-5p** |  |  | ✓ |
| 16 | **miR-342-3p** |  |  | ✓ |
| 17 | **miR-379-5p** | Up |  | ✓ |
| 18 | **miR-410-3p** | Up |  | ✓ |
| 19 | **miR-431-5p** | Up |  | ✓ |
| 20 | **miR-433** | Up |  | ✓ |
| 21 | **miR-487b-3p** | Up |  | ✓ |
| 22 | **miR-193b-3p** |  | Up |  |
| 23 | **miR-484** |  |  | ✓ |
| 24 | **miR-142-3p** |  | Down | ✓ |
| 25 | **miR-21-5p** |  |  |  |
| 26 | **miR-451a** |  | Down |  |
| 27 | **miR-133a** | Up | Up | ✓ |
| 28 | **miR-23a** |  |  | ✓ |
| 29 | **miR-199a-3p** |  |  | ✓ |
| 30 | **miR-185-5p** |  |  | ✓ |
| 31 | **miR-361-3p** |  |  | ✓ |
| 32 | **miR-361-5p** |  |  |  |
| 33 | **miR-98-5p** |  |  | ✓ |
| 34 | **miR-223-3p** |  |  | ✓ |

**References**

Jeanson-Leh, L. et al. (2014) ‘Serum Profiling Identifies Novel Muscle miRNA and Cardiomyopathy-Related miRNA Biomarkers in Golden Retriever Muscular Dystrophy Dogs and Duchenne Muscular Dystrophy Patients.’, The American journal of pathology, 184(11), pp. 2885–98. doi: 10.1016/j.ajpath.2014.07.021.

Vignier, N. et al. (2013) ‘Distinctive serum miRNA profile in mouse models of striated muscular pathologies.’, PloS one. Edited by T. Preiss. Public Library of Science, 8(2), p. e55281. doi: 10.1371/journal.pone.0055281.

**Supplemental figure 1:** Expression of the Dlk1-Dio3 miRNAs miR-379-5p, miR-410 and miR-431 in the *Cranialis tibial* and the cardiac left ventricle of a small cohort (n=2 or 3) GRMD and control dogs. Of note, in addition to miR-379, two other DLK1-DIO3 miRNAs were quantified. Both miR-431 and miR-410 showed a trend toward upregulation in the Cranial Tibialis biopsies of the moderate, but not the severe GRMD. In the cardiac samples, miR-433 was undetected, while as miR-379 and miR-410 were not dysregulated

.


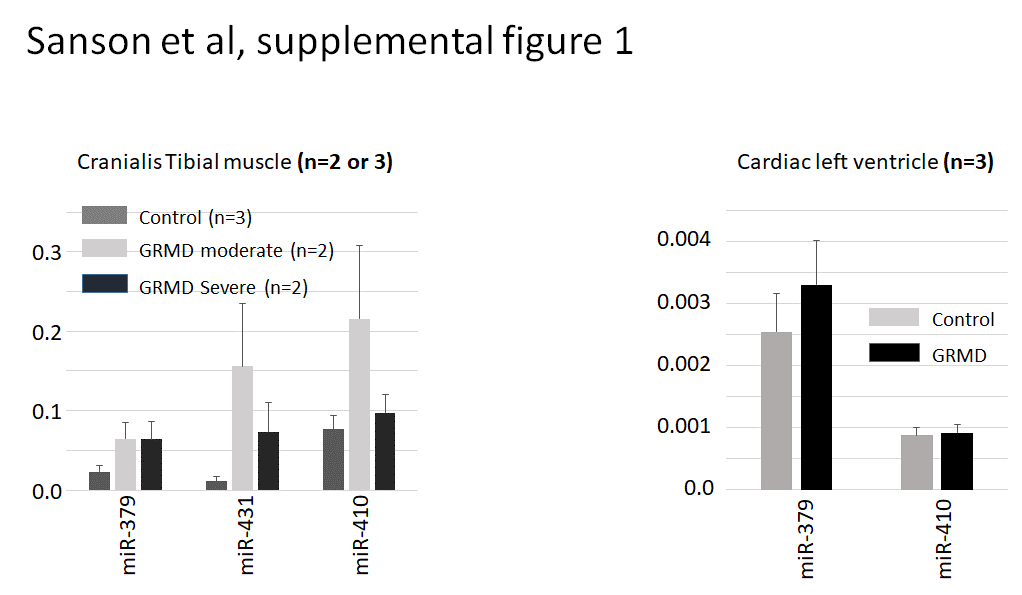


**Supplemental figure 2:** TargetScan predictions for miR-139 binding site on human EIF4G2 3’ UTR


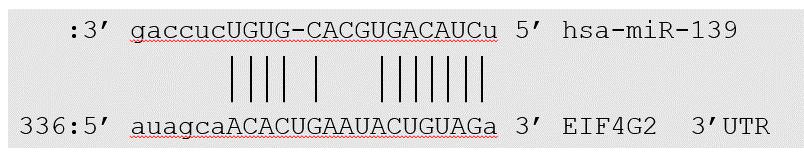


**Supplemental figure 3:** *In vitro* myotubes differentiation from sorted satellite in mdx and control mice

**Supplemental information: raw images for blots and gels**
